# T Cell Glycoengineering to Modulate Immune‐Tumor Crosstalk: A Universal Non‐Genetic Strategy for Enhanced Tumor Immunotherapy

**DOI:** 10.1002/advs.202505387

**Published:** 2025-12-08

**Authors:** Lihua Yao, He Yang, Fangjian Shan, Xiaomeng Niu, Yichen Wang, Hengyuan Zhang, Sujian Wang, Gaojian Chen, Hong Chen

**Affiliations:** ^1^ State Key Laboratory of Bioinspired Interfacial Materials Science State and Local Joint Engineering Laboratory for Novel Functional Polymeric Materials College of Chemistry Chemical Engineering and Materials Science Soochow University Suzhou 215123 P. R. China; ^2^ Jiangsu Biosurf Biotech Co., Ltd. Suzhou 215123 P. R. China

**Keywords:** biomedical polymer material, cell surface modification, click chemistry, glycopolymer, tumor immunotherapy

## Abstract

Gene‐engineered T cell therapies, particularly chimeric antigen receptor (CAR)‐T cells, have demonstrated remarkable clinical success. However, concerns regarding insertional mutagenesis and other risks associated with genetic modification remain. Here, a non‐genetic strategy is presented for T cell engineering using glycopolymer modification. It develops glycopolymer‐modified T (G‐T) cells based on antigen‐specific T cells by integrating metabolic glycoengineering and click chemistry, yielding cells that retain T cell functionality while significantly enhancing tumor enrichment. The polyvalent glycopolymer‐receptor interactions significantly improved the binding affinity of G‐T cells to various glucose transporter 1 (GLUT1)‐overexpressing tumor cells, resulting in increased cytotoxicity compared to unmodified T cells. In the tumor microenvironment, G‐T cells engaged in stronger immune crosstalk with dendritic cells (DCs), upregulating interferon‐gamma (IFN‐γ) and interleukin‐12 (IL‐12) secretion and amplifying the anti‐tumor immune response. Notably, despite the lower specificity of glycan–receptor interactions compared to antigen–antibody binding, the findings reveal an unexpected advantage: the “less restrictive” nature of glycan–receptor recognition enhances both tumor and immune cell interactions, triggering a potent immune cascade. This study establishes a universal, non‐genetic T cell engineering strategy with broad applicability, offering a new perspective for tumor immunotherapy by merging biomedical polymer materials with immune modulation.

## Graphical abstract

This study describes a T cell surface engineering strategy that integrates polymer materials with tumor immunology, aiming to achieve broad‐spectrum anti‐tumor applications of glycopolymer‐engineered T (G‐T) cells via non‐genetic modification. Glycopolymer engineering generally modulates immune‐tumor crosstalk through specific cell‐cell interactions mediated by glycopolymers, thereby enhancing the anti‐tumor immunity of G‐T cells.

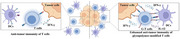

## 1 Introduction

T cell‐based immunotherapy has revolutionized tumor treatment by leveraging the intrinsic ability of T lymphocytes to recognize and eliminate malignant cells.^[^ ^1^, ^2^ ^]^ Despite the clinical success of chimeric antigen receptor (CAR) T cell therapies (for example, >80% overall response rate in B‐cell Acute Lymphoblastic Leukemia),^[^ ^3^, ^4^ ^]^ their reliance on genetic engineering poses inherent limitations,^[^ ^5^ ^]^ including potential safety concerns such as gene silencing, overexpression, or mutations induced by viral vectors,^[^ ^6^ ^]^ as well as off‐target mutations associated with CRISPR/Cas9 technology.^[^ ^7^ ^]^ These challenges underscore an urgent need for non‐genetic engineering strategies capable of endowing T cells with potential broad‐spectrum tumor‐targeting capabilities while preserving cellular safety and scalability.^[^ ^8^ ^]^

Recent advances in merging chemistry science with immuno‐engineering have opened new avenues for cell surface engineering.^[^ ^9^, ^10^, ^11^, ^12^, ^13^ ^]^ Gong et al. covalently attached polyethylene glycol (PEG) to the surface of CAR T cells to create a polymeric spacer, thereby modulating the intercellular interactions between CAR T cells, monocytes, and tumor cells, effectively alleviating cytokine release syndrome and neurotoxicity.^[^ ^14^ ^]^ Notably, glycan‐receptor interactions, as a cornerstone of biological recognition processes, offer unique opportunities for modulating cell‐cell interactions.^[^ ^15^, ^16^ ^]^ In contrast to the complex and heterogeneous structures of natural glycans, synthetic glycopolymers offer advantages such as simple synthesis, well‐defined structures, and tunable physicochemical properties, making them excellent candidates for cell surface glyco‐engineering research.^[^ ^17^, ^18^, ^19^, ^20^ ^]^ Moreover, studies have shown that glucose transporters (GLUT), such as GLUT1, are highly expressed on the surface of various tumor cells, aiding in the uptake of glucose nutrients.^[^ ^21^ ^]^ This provides new opportunities for designing immune therapeutic strategies that target sugar receptors as universal markers for tumors.^[^ ^22^, ^23^ ^]^

**Scheme 1:**
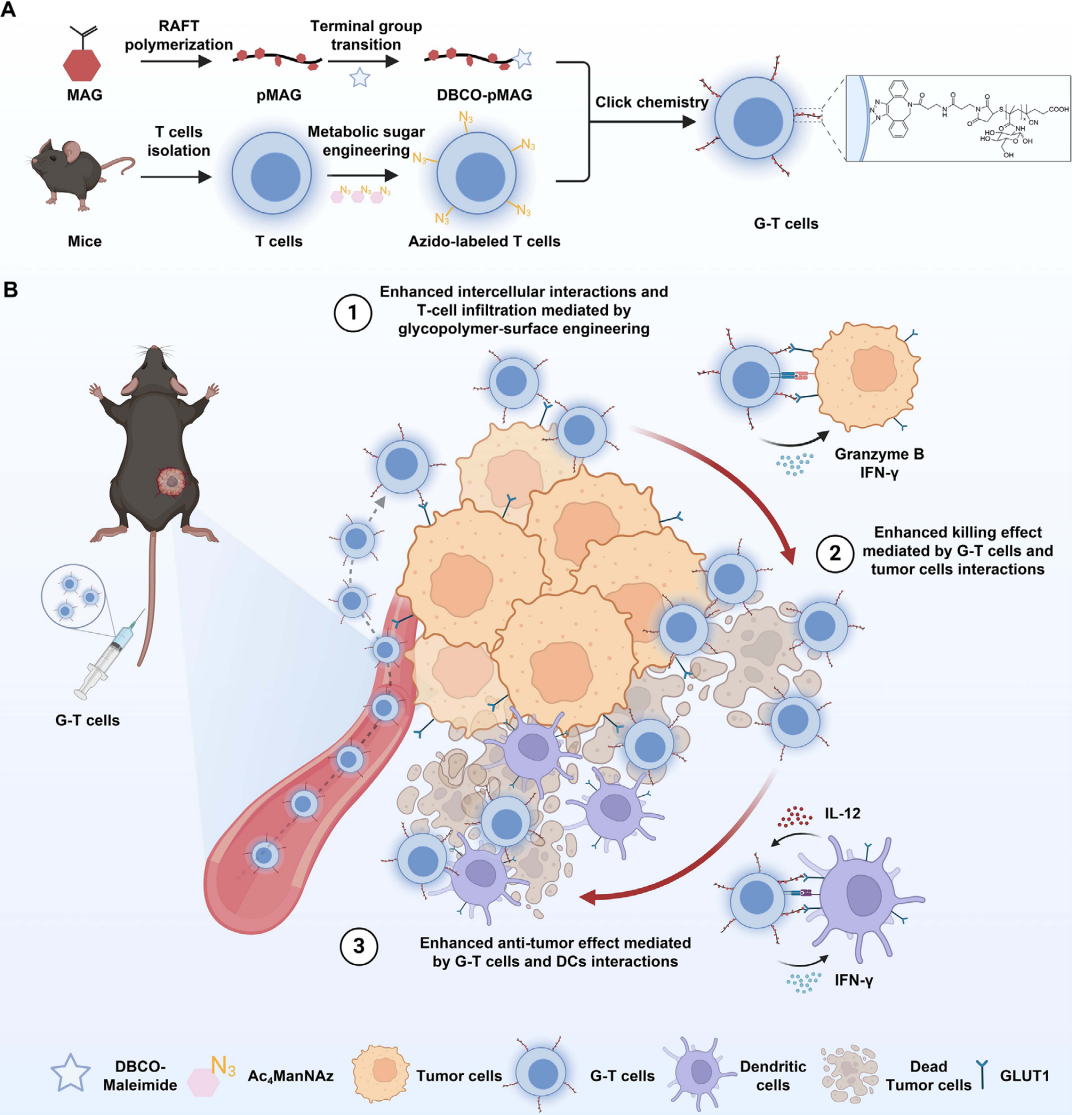
Schematic diagram of construction and application of glycopolymer‐engineered T cells. A) The glycopolymers were synthesized via RAFT polymerization followed by terminal‐group transition, and subsequently functionalized on the surface of T cells using metabolic labeling and click chemistry techniques. B) The interaction between the glycopolymer on the surface of G‐T cells and the highly expressed GLUT1 on tumor cells enhances the binding between T cells and tumor cells, thereby increasing the cytotoxic efficacy of G‐T cells. Additionally, the adhesion between G‐T cells and GLUT1‐overexpressing dendritic cells is augmented, promoting IL‐12 secretion and further enhancing the anti‐tumor activity of G‐T cells.

Herein, we present a chemical strategy to engineer antigen‐specific T cells with tumor‐targeting glycopolymers via metabolic labeling and bioorthogonal click chemistry (**Scheme** **1**). Inspired by the multivalent glycan‐receptor recognition paradigm in nature, we hypothesized that glycopolymers could amplify T cell‐tumor interactions through polyvalent binding while bypassing genetic modification. To validate this, we first synthesized dibenzocyclooctyne‐terminated glycopolymers (DBCO‐pMAG) via reversible addition‐fragmentation chain transfer (RAFT) polymerization combined with terminal group transition, and then modified onto T cells utilizing copper‐free click‐chemistry in combination with metabolic glycoengineering to construct glycopolymer‐engineered T cells (G‐T cells). Cell tracking imaging showed that polyvalent binding between the glycopolymer and GLUT1 significantly enhanced the interaction between G‐T cells and tumor cells overexpressing GLUT1. In vitro co‐incubation assays demonstrated that this enhanced interaction increased the cytotoxicity of T cells against various tumor cells by 34% to 47%. Moreover, we unexpectedly observed that the glycopolymer also facilitated enhanced interactions between dendritic cells (DCs) and T cells, likely due to the widespread high expression of GLUT1 on the surface of DCs. The strengthened crosstalk between DCs and G‐T cells led to an upregulation in DCs secretion of interleukin (IL)‐12, thereby further augmenting the antitumor activity of G‐T cells. Adoptive transfer of G‐T cells into B16‐OVA (OVA antigen‐expressing) tumor models resulted in effective anti‐tumor responses, attributed to increased T cell enrichment and upregulation of DCs maturation within the tumor microenvironment. This strategy was validated across multiple tumor models and achieved a 50% complete tumor remission rate when combined with immune checkpoint therapy. By integrating chemical and immunoengineering perspectives, this work establishes a versatile, non‐genetic platform for antigen‐specific T cell surface engineering. The modularity of this system‐compatible with diverse glycan motifs and tumor cell types‐positions it as a novel strategy for next‐generation tumor immunotherapy.

## 2 Results and Discussion

### 2.1 Construction of G‐T Cells via Metabolic Labeling Combined with Click Chemistry

**Figure 1:**
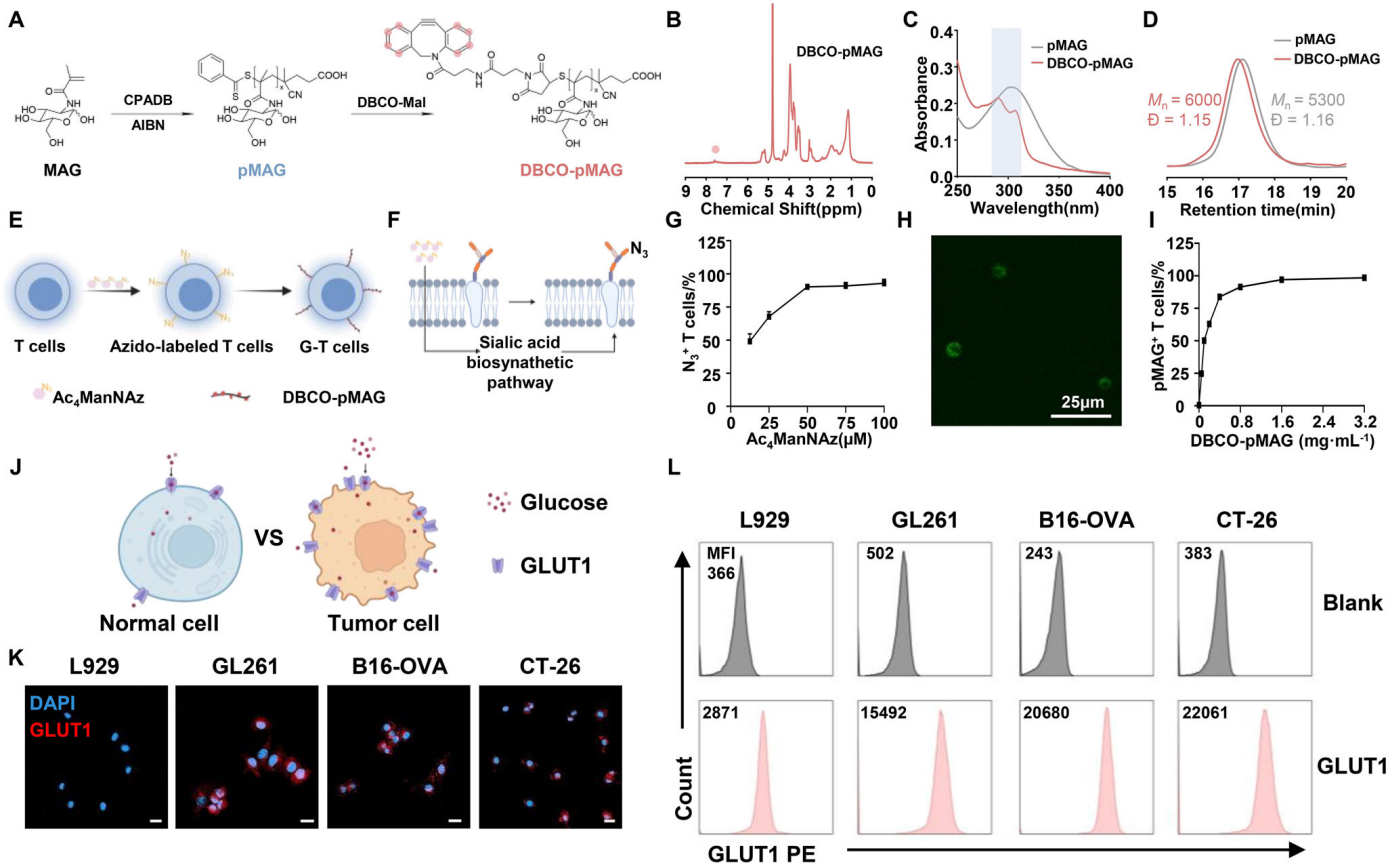
Construction of glycopolymer‐engineered T cells and investigation of GLUT1 expression on tumor cells. A) The synthesis route of DBCO‐pMAG. B) ^1^H NMR spectrum of DBCO‐pMAG. (Red dots reprensent H atoms on the benzene ring) C) UV–vis spectrum of pMAG and DBCO‐pMAG. D) SEC traces of pMAG and DBCO‐pMAG. E) Diagram illustrating the generation of G‐T cells. F) Schematic diagram of metabolic labeling of T cells with Ac_4_ManNAz. G) Percentage of azido^+^ T cells with different concentrations of Ac_4_ManNAz. H) CLSM images of G‐T cells. Scale bar = 25 µm. I) Percentage of pMAG^+^ T cells with various concentrations of DBCO‐p(MAG‐*co*‐FITC). J) Diagram of GLUT1 expression on the surface of normal and tumor cells. K) Representative CLSM images and L) flow cytometry statistics showing the expression of GLUT1 on L929, GL261, B16‐OVA, and CT‐26 cells (Mean Fluorescence Intensity is abbreviated as MFI.) Scale bar = 25 µm. The error bars represent mean ± SD (n = 3).

Considering the limitations of genetic approaches, we employed a chemical engineering strategy, metabolic labeling combined with click chemistry to construct glycopolymer‐engineered T cells (G‐T cells). Our approach involved synthesizing a click‐reactive glycopolymer and subsequently conjugating it to metabolically labeled T cells. The glycopolymer, designed for terminal‐group click reactivity, was synthesized via RAFT polymerization. This method allowed for precise control over the average polymer chain length and ensured high fidelity of the terminal end‐groups for subsequent modification. Specifically, we used 2,2′‐azoisobutyronitrile (AIBN) as the initiator, 4‐cyanopentanoic acid dithiobenzoate (CPADB) as the chain transfer agent, and 2‐methacrylamido glucopyranose (MAG) as the monomer (Figures S1–S3, Supporting Information).^[^ ^24^ ^]^ Following RAFT polymerization, we performed terminal‐group transition to yield DBCO‐functionalized glycopolymer (DBCO‐pMAG) (**Figure** **1**A). The successful synthesis of DBCO‐pMAG was confirmed by distinct signals in the ^1^H NMR spectrum at 7‐8 ppm (Figure 1B) and the appearance of a new UV absorption peak at 283–382 nm (Figure 1C). Furthermore, a notable increase in molecular weight alongside a narrow molecular weight distribution for the synthesized polymer was observed (Figure 1D).

*N*‐azidoacetylmannosamine‐tetraacylated (Ac_4_ManNAz), a widely utilized metabolic labeling agent, introduces unnatural azido (‐N_3_) groups onto the surface of living cells and enables subsequent bioorthogonal coupling with other functional groups to cell surfaces (Figure 1E).^[^ ^25^, ^26^ ^]^ After a 3‐day incubation with Ac_4_ManNAz (25–100 µm), the incorporation of azido groups on the T cell surface was confirmed by flow cytometry (Figure 1F). The results showed a concentration‐dependent labeling, with more than 90% of the cells exhibiting azido incorporation at 50 µM Ac_4_ManNAz (Figure 1G). Meanwhile, the azido‐labeled T cells (N_3_‐T cells) exhibited no significant cytotoxicity at the tested concentrations range (Figure S4, Supporting Information). Thus, a concentration of 50 µm was selected in subsequent experiments to construct G‐T cells. Subsequently, fluorescein isothiocyanate (FITC) labeled glycopolymers (DBCO‐p(MAG‐*co*‐FITC)) were synthesized to evaluate the construction of G‐T cells (Figures S5–S7, Supporting Information). Confocal laser scanning microscope (CLSM) images showed that the characteristic fluorescence of FITC was primarily localized on the T cell membrane, confirming the uniform decoration of glycopolymers on the T cell surface (Figure 1H). As expected, the conjugation efficiency via click chemistry exhibited a dose‐dependent relationship, with higher concentrations of DBCO‐pMAG resulting in an increased percentage of fluorescence on the cell surface within a defined concentration range until the plateau 0.8mg mL^−1^ (Figure 1I). Importantly, glycopolymer modification did not significantly affect T cell survival, proliferation, phenotype, aberrant activation or premature exhaustion (Figures S8–S11, Supporting Information).

Previous studies have shown that tumor cells achieve high glycolytic activity by upregulating glucose transporters (Figure 1J).^[^ ^27^ ^]^ We selected several commonly used mouse tumor models, including melanoma expressing the OVA antigen (B16‐OVA), glioma (GL261), and colon carcinoma (CT‐26),^[^ ^28^ ^]^ to assess GLUT1 expression on their surfaces using CLSM and flow cytometry, with normal mouse fibroblast cells (L929) serving as the control. CLSM images exhibited that GLUT1 expression on tumor cells was found to be significantly higher than on normal cells (Figure 1K). Flow cytometry analysis revealed that the expression of GLUT1 on the three tumor cell types was approximately 5.9‐8.0 times higher compared to L929 cells. (Figure 1L; Figure S11, Supporting Information). This upregulation of GLUT1 presents a promising target for G‐T immunotherapy strategies aimed at tumors that overexpress GLUT1.

### 2.2 Investigation of Contact Behavior Between G‐T Cells and Tumor Cells

**Figure 2:**
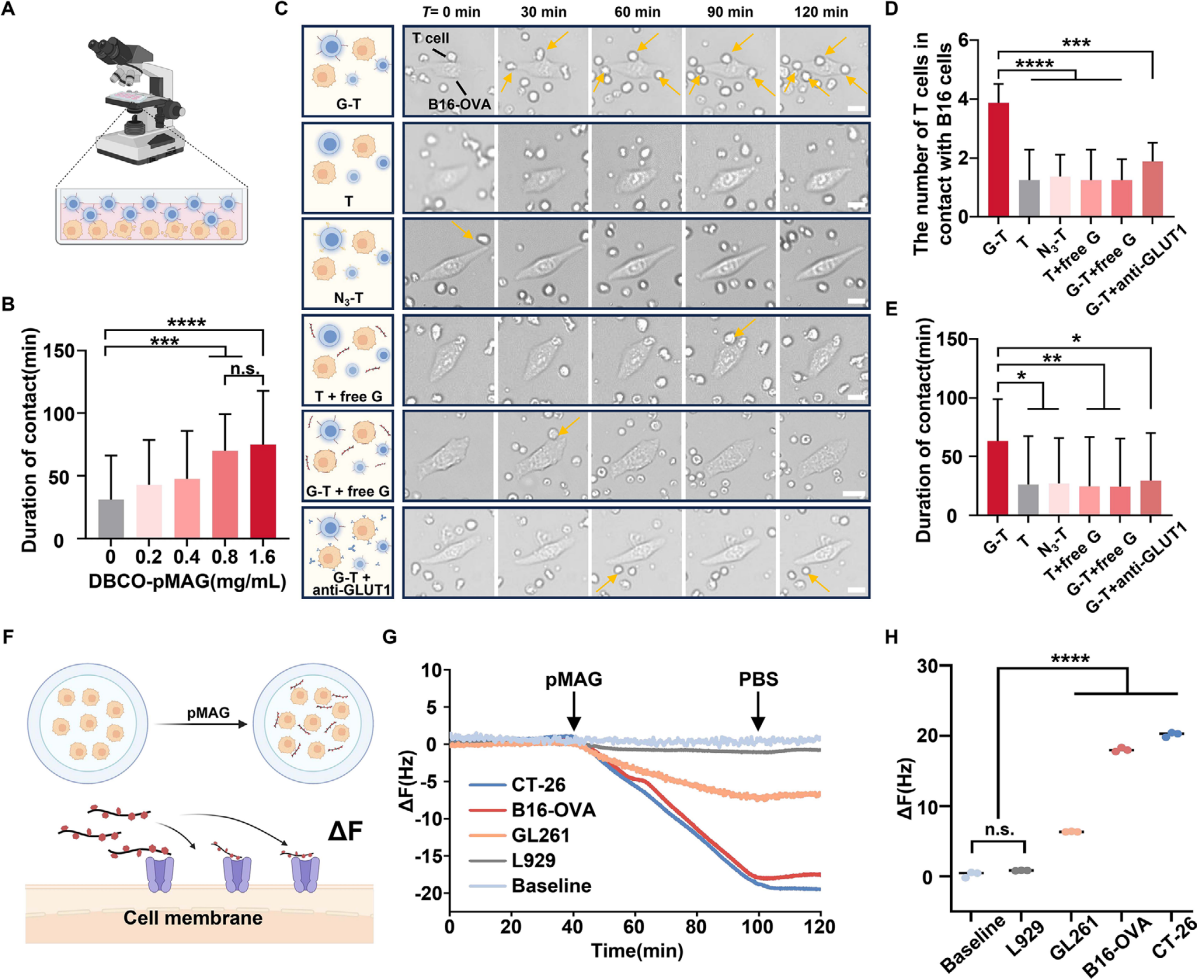
Investigation of contact behavior and underlying mechanism between G‐T cells and B16‐OVA cells. A) Schematic diagram of the interaction between G‐T cells and B16‐OVA cells. B) Duration of contact between G‐T cell and B16‐OVA cells at different concentrations of DBCO‐pMAG, with G‐T cell number (n = 30). C) Image tracking of T cells and B16‐OVA cells migration over time at 10:1 E/T ratio. Scale bar = 10 µm. D) The number of T cells in contact with B16‐OVA cells, with B16‐OVA cells number (n = 8). E) Duration of contact between T cell and B16‐OVA cells, with T cell number (n = 25). G‐T: glycopolymer‐engineered T cells; T: untreated T cells; N_3_‐T: azido‐labeled T cells; T+free G: T cells and free glycopolymers in the cell culture medium; G‐T+free G: glycopolymer‐engineered T cells and B16‐OVA cells modified with pMAG in the cell culture medium; G‐T+anti‐GLUT1: glycopolymer‐engineered T cells and B16‐OVA cells modified with GLUT1 inhibitor in the cell culture medium. F) Schematic diagram of the interaction between glycopolymers and tumor cells tested by QCM. G) The frequency curve of glycopolymers binding to chips preincubated with cells. H) ΔF caused by binding of glycopolymers chips preincubated with cells. The error bars represent mean ± SD. Statistical analysis was performed using Tukey's multiple comparison test and, n.s.>0.05, *p < 0. 05, **p < 0.01, ***p < 0.001, ****p < 0.0001.

The ability of T cells recognizing and interacting with tumor cells is a cornerstone of anti‐tumor immunity.^[^ ^29^ ^]^ Enhancing these interactions is pivotal for improving the effectiveness of adoptive T cell therapies, especially in addressing challenges such as tumor heterogeneity and immune evasion.^[^ ^30^ ^]^ In this study, we explored the impact of glycopolymer modifications on T cell‐tumor cell interactions, with the aim of developing novel strategies to boost antigen‐specific T cell‐mediated tumor binding and elimination. Ovalbumin (OVA)‐specific T cells, isolated from OT‐1 transgenic mice, were used as antigen‐specific T cells, while B16‐OVA cells expressing the OVA antigen served as target cells for the subsequent experiments. To assess whether glycopolymer modifications could enhance T cell recognition of tumor cells, we conducted cell image tracking experiments to monitor the interactions between Ovalbumin (OVA)‐specific T cells, isolated from OT‐1 transgenic mice, and B16‐OVA cells at an effector‐to‐target ratio of 10:1 (**Figure** **2**A). The binding events between T cells and tumor cells were recorded every minute for 2 h, with T cells and tumor cells distinguishable by their size and morphology. A key factor influencing the intercellular interactions between G‐T cells and tumor cells was the concentration of glycopolymers. Cell tracking imaging of G‐T cells and tumor cells migration revealed that glycopolymers on the surface of G‐T cells enhanced the interaction between T cells and tumor cells. The strength of these interactions increased in a glycopolymer concentration‐dependent manner, plateauing at a concentration of 0.8 mg mL^−1^, indicating that glycopolymer surface modification approached saturation at this concentration (Figure 2B; Figure S14, Supporting Information).

Subsequently, we investigated the mechanism underlying the altered contact behavior between T cells and B16‐OVA cells. A significant increase in both the number of G‐T cells in contact with B16‐OVA cells and their adhesion duration was observed compared to T cells, with the number of G‐T cells increasing ≈3.1 times over 120 min, and the average adhesion time reaching 63 min, compared to 26 min for T cells (Figure 2C–E). No significant difference was found between T cells and N_3_‐T cells. Moreover, the presence of free glycopolymers in the co‐incubation system did not enhance neither the number nor duration of cell contact, further supporting the necessity of glycopolymer anchoring on T cell surfaces to promote effective interaction with tumor cells (Figure 2C–E). We also pre‐incubated B16‐OVA cells with glycopolymer or GLUT1 inhibitor before co‐incubating with T cells. The results revealed that blocking the GLUT1 receptors on the B16‐OVA cell surface abolished the enhanced interactions with G‐T cells (Figure 2C–E). These findings suggest that the increased contact between G‐T cells and tumor cells is mediated by specific interactions between the glycopolymers and glucose receptors, such as GLUT1, on the surface of the tumor cells.

To further investigate the molecular mechanism, we employed quartz crystal microbalance (QCM) to assess the impact of glycopolymers on different tumor cells. Various tumor cell lines with high GLUT1 expression, including GL261, B16‐OVA, and CT‐26, were stably incubated on the QCM chip surface (Figure 2F). We observed that upon passing glycopolymers over the chip, frequency shifts occurred due to the binding of glycopolymers to tumor cell surface receptors, such as GLUT1, with the frequency changes reaching a plateau as binding approached saturation. Notably, the maximum frequency change correlated positively with the GLUT1 expression levels on the tumor cell surfaces (Figure 2G,H; Figure S12, Supporting Information). In contrast, L929 cells, which express lowest levels of GLUT1, exhibited minimal frequency changes. To further substantiate the role of GLUT1 in this process, we generated GLUT1‐knockout B16‐OVA cells using a gene‐editing strategy (Figure S15A, Supporting Information). Notably, no statistically significant differences were observed between G‐T cells and unmodified T cells when GLUT1 expression was ablated, in sharp contrast to the markedly enhanced binding and cytotoxicity observed against wild‐type B16‐OVA cells (Figure S15B–D, Supporting Information). These findings provide compelling evidence that GLUT1 plays a pivotal role in enhancing the interactions between G‐T cells and tumor cells. Since tumor cells also express other sugar receptors, such as GLUT3, GLUT4 and sialic acid‐binding receptors,^[^ ^31^, ^32^ ^]^ whose expression profiles vary across tumor types, a more comprehensive mechanistic analysis is required in the following specialized studies. Nevertheless, our findings provide initial insights into the molecular mechanism underlying the interaction between G‐T cells and tumor cells: the glycopolymers on the G‐T cell surface specifically bind to high‐expressing sugar receptors, with GLUT1 being a key receptor in this process.

### 2.3 Evaluation of the Ex Vivo Anti‐Tumor Effect of G‐T Cells

**Figure 3:**
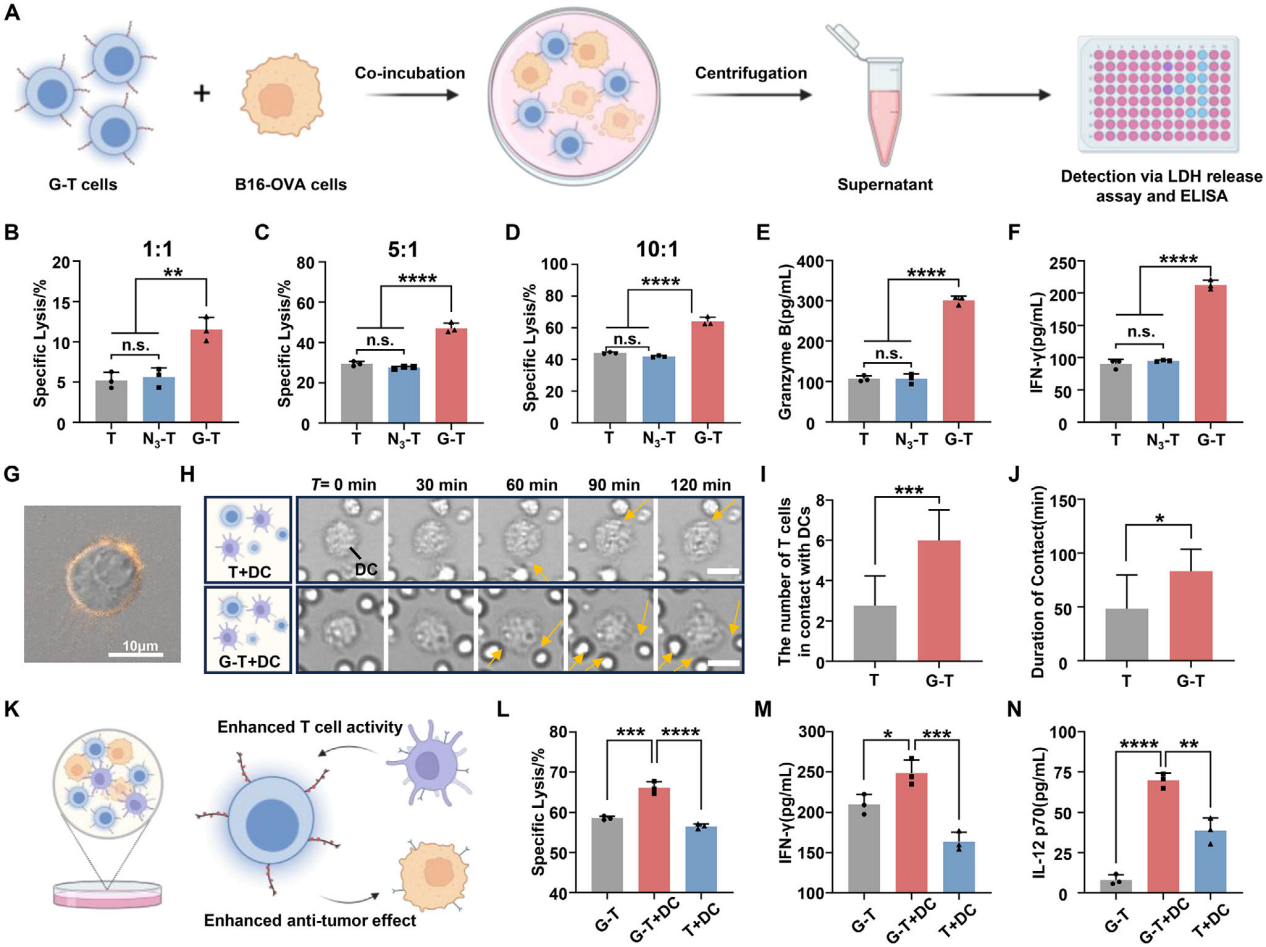
Anti‐tumor effect of G‐T cells ex vivo. A) Schematic diagram of the experimental procedures showing the antigen‐specific T cells killing B16‐OVA cells ex vivo. B) The lysis efficacies of B16‐OVA cells by T cells at the E/T ratios of 1:1, C)5:1, D)10:1. E) Cytokines secretion of Granzyme B and (F) IFN‐γ levels for T cells co‐incubated with tumor cells. G) Representative CLSM images of GLUT1 expression on the surface of DC. H) Image tracking of G‐T cells and DCs migration over time. Scale bar = 10 µm. I) The number of G‐T cells in contact with DCs, with DCs number (n = 8). J) Duration of contact between G‐T cells and DCs, with G‐T cells number (n = 8). K) Schematic diagram of interaction between DCs, G‐T cells and tumor cells. L) Lysis of tumor cells after incubation with G‐T cells and DCs. Cytokines secretion of M) IFN‐γ and N) IL‐12 p70 levels. The error bars represent mean ± SD (n = 3). Statistical analysis was performed using Tukey's multiple comparison test and, n.s. > 0.05, *p < 0. 05, **p < 0.01, ***p < 0.001, ****p < 0.0001.

Next, we investigated whether the enhanced intercellular interactions could serve as a novel strategy to augment the cytotoxic effects of G‐T cells on tumor cells (**Figure** **3**A). G‐T cells and B16‐OVA cells were co‐incubated at effector‐to‐target (E/T) ratios of 1:1, 5:1, and 10:1, and cytotoxicity was assessed using lactate dehydrogenase (LDH) release assays. The results demonstrated that G‐T cells were significantly more effective in eliminating B16‐OVA tumor cells compared to unmodified T cells, particularly at a 10:1 E/T ratio, with a 1.5‐fold increase in cytotoxicity (Figure 3B–D). Furthermore, the secretion of two key inflammatory cytokines, Granzyme B and interferon‐γ (IFN‐γ), by cytotoxic T cells was quantified using enzyme‐linked immunosorbent assays (ELISA), as they play a critical role in mediating tumor cell death. The data exhibited that G‐T cells secreted significantly higher levels of Granzyme B and IFN‐γ compared to unmodified T cells, with increases of 1.8‐fold and 1.4‐fold, respectively (Figure 3E,F). The increased secretion of inflammatory cytokines highlighted the enhanced anti‐tumor activity of glycopolymer‐modified T cells, suggesting that metabolic labeling and biorthogonal click chemistry‐mediated glycopolymer surface engineering could potentiate T cell cytotoxicity by improving their interactions with tumor cells.

Recent studies have shown that interactions between DCs and T cells can enhance T cell‐mediated anti‐tumor responses through the expression of co‐stimulatory molecules, such as CD86, and the secretion of pro‐inflammatory cytokines, such as IL‐12.^[^ ^33^, ^34^ ^]^ Additionally, DCs exhibit higher levels of GLUT1 expression on their surface compared to other immune cells (Figure 3G).^[^ ^35^ ^]^ Through cell tracking imaging experiments, we observed that the interaction between G‐T cells and DCs was significantly enhanced, both in terms of contact frequency and duration, compared to unmodified T cells (Figure 3H–J). Co‐incubation experiments involving DC‐G‐T cells‐tumor cells demonstrated that the presence of DCs further potentiated the tumor‐killing effects of G‐T cells, in contrast to unmodified T cells (Figure 3K,L). This enhancement was positively correlated with increased secretion of IFN‐γ in the culture supernatant (Figure 3M). Moreover, the crosstalk between G‐T cells and DCs facilitated the secretion of IL‐12 p70 by DCs (Figure 3N).^[^ ^36^ ^]^ Importantly, such enhancement in antitumor effect was substantially attenuated by the blockade of IL‐12 with a neutralizing antibody(Figure S17, Supporting Information). These findings showed that the crosstalk between G‐T cells and DCs further potentiated anti‐tumor cytotoxicity and IL‐12 secreted by DCs serves as a key mediator in the observed functional enhancement.

### 2.4 Tumor Immunotherapy by G‐T Cells In Vivo

Before evaluating the in vivo anti‐tumor efficacy of G‐T cells in mice, we first examined their tumor accumulation. T cells were labeled with the near‐infrared fluorescent dye DiR and injected intravenously into C57BL/6 mice bearing subcutaneous tumors (Figure S18A, Supporting Information). 24 h post‐injection, tumors were excised and subjected to fluorescence imaging. As shown in the fluorescence image (Figure S18B, Supporting Information), varying intensities of DiR signals were observed in tumor tissues following a single injection of either T or G‐T cells. Quantitative fluorescence analysis demonstrated a 22.9% increase in signal intensity within tumors of mice treated with G‐T cells relative to those receiving unmodified T cells (Figure S18C, Supporting Information), indicating enhanced intratumoral accumulation of glycopolymer‐engineered T cells, which can be attributed to the glycopolymer surface engineering. Such improvement was attributed to the enhanced adhesive interactions between G‐T cells and GLUT1‐overexpressing tumor cells, which may facilitate accumulation within the tumor vasculature. This effect provides a partial solution to the insufficient tumor infiltration, which is one of the key barriers in adoptive T cell therapy.

In vivo biodistribution of CFSE‐labeled cells revealed no significant differences between G‐T and unmodified T cells in heart, spleen, lung, or kidney, and only a modest increase in liver accumulation (Figure S19, Supporting Information). Corresponding histopathology showed no tissue injury (Figure S20, Supporting Information), and serum ALT, AST, BUN, and CREA remained within normal ranges (Figure S21, Supporting Information). These results collectively demonstrate that glycopolymer modification does not elicit systemic toxicity or off‐target accumulation, supporting the favorable biosafety profile of G‐T cells for in vivo application. Although GLUT1 is broadly expressed in normal tissues, its expression is markedly upregulated in malignant cells owing to enhanced glycolytic metabolism and tumor‐associated hypoxia, providing a relative rather than absolute molecular window for selective targeting. The glycopolymer modification described herein does not alter the intrinsic antigen specificity of T cells but instead reinforces intercellular adhesion through reversible GLUT1‐mediated interactions, thereby minimizing the risk of nonspecific cytotoxicity.

**Figure 4:**
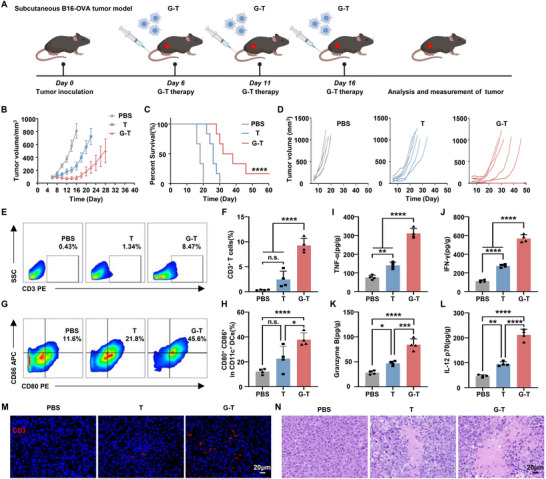
In vivo tumor immunotherapy by G‐T cells. A) Schematic diagram of G‐T cells treating mouse B16‐OVA tumor. B) Statistical tumor growth curves on B16‐OVA tumor‐bearing mice (n = 6). C) Survival rates of B16‐OVA tumor‐bearing mice. D) Individual tumor growth curves of B16‐OVA tumors on mice from different groups as indicated. E) Representative flow cytometry data showing the proportion of CD3^+^ T cells in tumor tissues. F) Statistic data corresponding to the flow cytometry data in (E). G) Representative flow cytometry data showing the proportion of CD80^+^CD86^+^ DCs in tumor tissues. H) Statistic data corresponding to the flow cytometry data in (G). I) TNF‐α, J) IFN‐γ, K) Granzyme B, and L) IL‐12 p70 levels in tumor tissues under different treatments as indicated. M) Immunofluorescence‐stained tumor slice images and N) H&E‐stained micrographs under different treatments. Scale bar = 20 µm. Survival curves were obtained using the Kaplan‐Meier method and compared by the log‐rank test. ****p<0.0001. The error bars represent mean ± standard deviations (n = 4). Statistical analysis was performed using Tukey's multiple comparison test and, n.s.>0.05, *p<0.05, **p<0.01, ***p<0.001,****p<0.0001.

As a proof of concept, G‐T cells were tested for in vivo immunotherapy in a B16‐OVA tumor model. To establish the melanoma model, B16‐OVA cells were subcutaneously injected into the backs of female C57BL/6 mice. Once the average tumor volume reached ≈100 mm^3^, the mice were randomly divided into three groups: 1) untreated group, 2) T cells group, and 3) G‐T cells group (**Figure** **4**A). G‐T cells were injected intravenously into tumor‐bearing mice on the 6th, 11th, and 16th day after tumor injection (Figure 4A). The tumor growth curves indicated that G‐T cells significantly enhanced the inhibition of tumor growth compared to T cells group (Figure 4B). Furthermore, G‐T cell treatment notably prolonged the survival of the mice, with complete tumor regression observed in one mouse (1/6) (Figure 4C). In addition, individual tumor growth curves revealed that mice treated with G‐T cells exhibited consistent tumor growth control during the treatment period (Figure 4D). However, tumor progression was observed in half of the mice (3/6) following the completion of therapy. Notably, one mouse achieved near‐complete tumor regression (1/6) by the end of treatment, while two others experienced sustained tumor growth inhibition for several days post‐treatment before tumor progression resumed. These findings suggested that G‐T cell therapy could elicit a systemic anti‐tumor immune response, albeit with variable outcomes among individual mice.

To investigate the underlying mechanisms responsible for the superior efficacy of G‐T cells, we conducted repeated tumor immunotherapy experiment. Upon completion of the treatment, mice were euthanized, and tumor tissues were collected for single‐cell suspension preparation and histological analysis. Flow cytometry results revealed that after three rounds of treatment, the T cell content in the tumor tissue of the G‐T cell group was approximately 3.8 times greater than that of the T cell group, indicating that glycopolymer surface modification effectively enhanced T cell targeting and enrichment into the tumor (Figure 4E,F). Additionally, the maturation of dendritic cells in the tumor tissue of the G‐T cell group was significantly increased (Figure 4G,H). ELISA data showed that, compared to the T cell group, the secretion levels of key inflammatory cytokines, such as TNF‐α, IFN‐γ, and Granzyme B, were elevated by 120%, 120% and 80%, respectively, likely due to the enhanced interaction between T cells and tumor cells (Figure 4I–K). We also measured the IL‐12 p70 levels in tumor tissues, a cytokine primarily secreted by mature dendritic cells. Consistent with our in vitro co‐incubation findings, IL‐12 p70 concentrations in the G‐T cell group were significantly higher than those in the T cell group, further supporting the enhanced tumor‐killing activity of T cells (Figure 4L). Representative immunofluorescence staining images revealed a marked increase in the number of CD3^+^ T cells (red) in the tumor tissue of the G‐T cell group (Figure 4M). Additionally, dendritic cell signals (green) were observed surrounding some T cells (Figure S22, Supporting Information). H&E staining further demonstrated significant tumor cell apoptosis and necrosis following G‐T cell treatment (Figure 4N). Based on these observations, we propose that G‐T cells, through glycopolymer‐mediated intercellular recognition and interaction, facilitate enhanced targeting and enrichment of T cells into tumors, thereby augmenting their cytotoxic activity. Furthermore, the apoptosis and necrosis of tumor cells likely promoted the recruitment of dendritic cells to the tumor site, where interactions with G‐T cells upregulated IL‐12 secretion. This cascade effect further potentiated the anti‐tumor activity of G‐T cells, resulting in a significantly stronger tumor suppression compared to unmodified T cells.

### 2.5 Tumor Immunotherapy by G‐T Cells Combined with Immune Checkpoint Blockade Therapy

**Figure 5:**
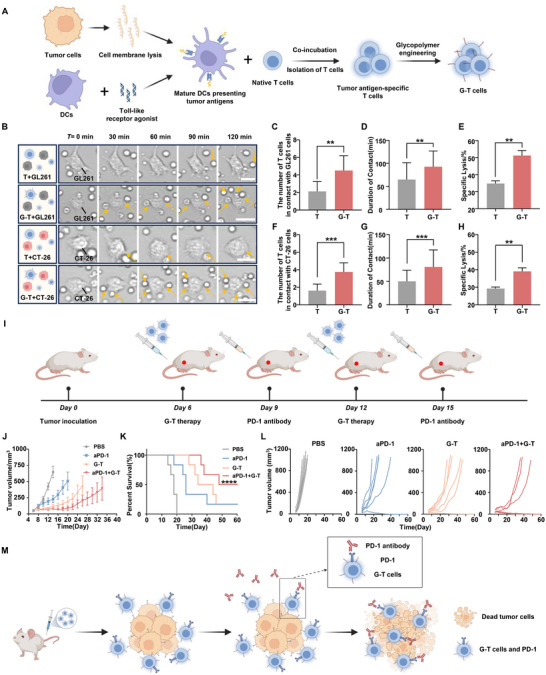
Universal exploration of G‐T cells and tumor immunotherapy combined PD‐1 antibody in CT‐26 tumor model. A) Construction of antigen‐specific G‐T cells for CT‐26 cells or GL261 cells. B) Image tracking of G‐T cells and GL261 or CT‐26 cells migration over time. Scale bar = 10 µm. C) Number of G‐T cells in contact with GL261 cells, with GL261 cells number (n = 8). D) Duration of contact between G‐T cells and GL261 cells, with G‐T cells number (n = 25). E) Lysis efficacies of GL261 cells by G‐T cells. F) Number of G‐T cells in contact with CT‐26 cells, with CT‐26 cells number (n = 8). G) Duration of contact between G‐T cells and CT‐26 cells, with G‐T cells number (n = 25). H) Lysis efficacies of CT‐26 cells by G‐T cells. I) Schematic diagram of G‐T cells combined with PD‐1 antibody to treat mouse colon carcinoma. J) Statistical tumor growth curves, K) survival rates and L) individual tumor growth curves of CT‐26 tumors on mice from different groups as indicated (n = 6). M) Diagram illustrating the mechanism underlying the enhancement of G‐T cell therapy by PD‐1 blockade. Survival curves were obtained using the Kaplan‐Meier method and compared by the log‐rank test. ****p < 0.0001. The error bars represent mean ± SD (n = 3). Statistical analysis was performed using Tukey's multiple comparison test and, **p < 0.01, ***p < 0.001.

To evaluate the therapeutic potential of G‐T cells across various tumor types, antigen‐specific G‐T cells for mouse colon carcinoma (CT‐26) or mouse glioma (GL261) were generated, both of which overexpress GLUT1, as outlined in **Figure** **5**A. Cell tracking imaging and LDH release assays revealed that glycopolymer surface modification significantly enhanced G‐T cell‐tumor cell interactions in both GL261 and CT‐26 models, thereby increasing the cytotoxic efficiency of G‐T cells against tumors by 47% and 34%, respectively (Figure 5B–H). One of the major obstacles in T cell therapy is the immunosuppressive tumor microenvironment. Recently, immune checkpoint blockade, particularly with PD‐1 inhibitors, has demonstrated promising therapeutic outcomes.^[^ ^37^, ^38^ ^]^ To further enhance the efficacy of G‐T cell therapy and assess its potential across different tumor types in vivo, we established a CT‐26 subcutaneous tumor model on BALB/c mice. G‐T cells were intravenously injected on the 6th and 12th days after tumor inoculation, while PD‐1 antibody was administered on the 9th and 15th days (Figure 5I). The average tumor volume curves indicated that the combination of G‐T cells and PD‐1 antibody significantly inhibited tumor growth compared to single treatment (Figure 5J). G‐T cells or PD‐1 antibody alone achieved a complete tumor remission rate of 1/6, while the combination treatment resulted in a 50% complete remission rate (3/6) (Figure 5K,L). This improvement is attributed to the PD‐1 antibody binding to PD‐1 on T cells, preventing its interaction with the PD‐1 ligand (PD‐L1) on tumor cells, thereby partially reversing the immunosuppressive effects of the tumor microenvironment and enhancing T cell‐mediated anti‐tumor responses (Figure 5M). These findings suggest that G‐T cells exhibit enhanced anti‐tumor activity across multiple tumor models, and their therapeutic efficacy can be further potentiated when combined with immune checkpoint inhibitors. From a translational standpoint, this chemically defined, non‐genetic engineering platform could be readily integrated with existing adoptive T‐cell therapy to augment tumor retention and functional persistence without compromising safety. Further evaluation using primary human T cells and humanized tumor models will be critical to delineate the therapeutic index and validate its clinical feasibility.

## 3 Conclusion

In summary, we demonstrate that glycopolymer‐based surface engineering of T cells significantly enhances their binding affinity and cytotoxic activity toward GLUT1‐overexpressing tumor cells, thereby improving the therapeutic efficacy of antigen‐specific T cell‐based tumor immunotherapy. In vivo experiments across multiple tumor models demonstrated that G‐T cells could efficiently inhibit the tumor growth and prolong survival. In addition, G‐T cells treatment notably increased interleukin (IL)‐12 levels, likely resulting from crosstalk between G‐T cells and DCs, which triggered a cascade of immune activation and further enhanced the anti‐tumor immune responses. Moreover, combination with PD‐1 blockade synergistically potentiated anti‐tumor efficacy, resulting in a notable complete remission rate of 50%. Future studies should investigate the precise mechanisms by which glycopolymers modulate tumor immune crosstalk, incorporating gene silencing and genetic analyses for a deeper understanding. Collectively, this work establishes a glycopolymer‐based strategy for T cell engineering as a non‐genetic and broadly applicable immunotherapeutic platform, bridging biomedical polymer design with immune modulation and offering new opportunities for next‐generation tumor immunotherapy.

## 4 Experimental Section

### Chemical and Biochemical Reagents

*N*‐azidoacetylmannosamine‐tetraacylated (Ac_4_ManNAz), fluorescein O‐methacrylate (FluMA), and Cy5‐conjugated dibenzylcyclooctyne (DBCO‐Cy5) were purchased from Sigma‐Aldrich Chemical Co., (Shanghai, China). D‐glucosamine hydrochloride and DBCO‐maleimide were purchased from TCI Co., (Shanghai, China). Triethylamine, ethanolamine, and dimethyl sulfoxide (DMSO) were purchased from Aladdin (Shanghai, China). Potassium carbonate (K_2_CO_3_), *N*,*N*‐Dimethylformamide (DMF), 2,2′‐azoisobutyronitrile (AIBN), magnesium sulfate and methanol were purchased from Sinopharm Chemical Reagent Co. (Shanghai, China) and AIBN was recrystallized from ethanol and dried under vacuum before use. 4‐Cyanopentanoic acid dithiobenzoate (CPADB) was purchased from Sigma‐Aldrich. Lactate dehydrogenase (LDH) cytotoxicity assay kit was purchased from Beyotime Biotechnology. Granzyme B, TNF‐α, IFN‐γ, and interleukin (IL)‐12 p70 enzyme‐linked immunosorbent assay (ELISA) kit, bicinchoninic acid protein (BCA) assay kit were purchased from Elabscience Biotechnology Co. Ltd. Recombinant murine interleukin‐2 (IL‐2) was purchased from Solarbio (Beijing, China). Cytidylyl phosphate guanosine (CpG) was purchased from Sangon Biotech. Dynabeads^TM^ mouse T cell‐Activator CD3/CD28 (cat. No. 11452D) were purchased from Gibco. MojoSort™ Mouse CD3 T Cell Isolation Kit and flow antibodies including PE anti‐mouse CD3 antibody (anti‐CD3‐PE, cat. No.100205), PerCP anti‐mouse CD8a antibody (anti‐CD8a‐PerCP, cat. No.100731), FITC anti‐mouse CD11c antibody (anti‐CD11c‐FITC, cat. No.117306), PE anti‐mouse CD80 antibody (anti‐CD80‐PE, cat. No.104708), APC anti‐mouse CD86 antibody (anti‐CD86‐APC, cat. No.105012) were purchased from Biolegend. Roswell Park Memorial Institute (RPMI) 1640 medium, fetal bovine serum (FBS), and penicillin‐streptomycin (P/S) were purchased from Gibco (Grand Island, NY, USA). MEM medium was purchased from Biosharp (Anhui, China). Plasmocin™ was obtained from Invivogen Co., Ltd (San Diego). Deionized water (DIW), purified to a minimum resistivity of 18.2 MΩ·cm by a Millipore water purification system, was used in all experiments.

### Cell Lines and Animals

CT‐26 cells (Cell Bank, Shanghai Institutes for Biological Sciences, Chinese Academy of Sciences), and B16‐OVA cells (a gift from Z. Liu, Soochow University) were cultured in RPMI 1640 medium. GL261 cells (Cell Bank, Shanghai Institutes for Biological Sciences, Chinese Academy of Sciences) were cultured in DMEM medium with high glucose. Fibroblasts (L929) (Enzyme Research Biotechnology Co., Ltd (Shanghai, China)) were cultured in MEM medium. Both mediums contain 10% FBS, 100 U mL^−1^ penicillin, 100 µg mL^−1^ streptomycin and 5 µg mL^−1^ Plasmocin^TM^ prophylactic at 37 °C in 5% CO_2_. In addition, the culture medium for all cells lines were refreshed every 1‐2 days. Female C57BL/6 mice, BALB/c mice, and OT‐1 mice (6‐8 weeks) were purchased from the Laboratory Animal Center of Soochow University. All animal experiments complied with the animal protection laws of China and were conducted by following the protocols approved by the Institutional Animal Care and Use Committee of Soochow University (approval number: No. ECSU‐2019000198).

### Characterization

^1^H NMR spectra were recorded on either a 300 MHz Vnmrs or 400 MHz Bruker Avance Neo. Chemical shifts are reported in parts per million (ppm) with respect to the residual solvent peak (D_2_O, 4.79 ppm for ^1^H NMR). Number‐average molecular weights (*M*n) and polydispersities (PDI) of the polymers were determined by a gel permeation chromatography (GPC) Waters 1515 (Waters, USA) system equipped with a refractive‐index detector, using Ultrahydrogel 6 × 40 mm Guard Column at 35 °C (water as the mobile phase consisting of 0.2 M NaNO_3_ and 0.01 M NaH_2_PO_4_, adjusted to pH = 9, flow rate 1 mL min^−1^). The UV–vis spectrum of glycopolymers were obtained by Shimadzu UV‐3600. The multifunctional microplate reader (Varioskan flash, Thermo Science) was used to evaluate cell viability. Flow cytometry was performed on a BD FACS Verse system. The Video of T cells interaction with tumor cells were observed by using inverted fluorescence microscopy (Nikon Eclipse Ti‐S, Japan). The glycopolymer‐engineered T cells were observed with a laser confocal microscopy (Leica DMI6000 CS, USA).

### Synthesis of Glycopolymers Containing DBCO Terminal Group (DBCO‐pMAG)

Glycopolymer monomers, glucose monomers (MAG), were synthesized as reported previously.^[^ ^24^ ^]^ Glycopolymers were synthesized through reversible additional‐fragmentation chain transfer (RAFT) polymerization with CPADB as the chain transfer agent and AIBN as the initiator. In brief, MAG (0.74 g, 3.0 mmol), CPADB (0.017 g, 0.060 mmol), and AIBN (0.0056 g, 0.030 mmol) were dissolved in 1 mL DMSO solvent. The polymerization was carried out at 70 °C for 12 h after deoxygenation by bubbling with argon for 30 min. The polymer solution was then dialyzed against DIW for 3 days and then lyophilized to obtain pink solid products pMAG. Triethylamine (2 µL), excess ethanolamine (100 µL), and DMF solution with DBCO‐maleimide (2 mg) were added to pMAG solution (30 mg, 1 mL) dissolved in DIW. The reaction was carried out at 25 °C for 18 h after deoxygenation by bubbling with argon for 15 min. The reaction mixture was dialyzed for 3 days against deionized water and then lyophilized to give white solid products DBCO‐pMAG.

### T Cell Isolation, Activation, and Expansion

To isolate native T cells and OVA‐specific T‐cells, spleen was excised from C57BL/6 mice and OT‐1 mice. Then, the excised spleen was ground through a 40 µm size cell strainer. The cell suspensions were centrifuged at 1500 revolutions per minute (rpm) for 3 min and incubated with 1×red blood cell (RBC) lysis buffer for 5 min with occasional shaking. Then, 3 ml of PBS was added to the cell suspension to stop the lysis process. The splenocytes were obtained by centrifugation (1200 rpm, 3 min) and resuspended in 2 mL RPMI medium for subsequent use. T cells were isolated following the protocol of MojoSort Mouse CD3 T Cell Isolation Kit. T cells were activated by Dynabeads mouse T‐Activator CD3/CD28 in RPMI 1640 medium containing 10% fetal bovine serum, 1% penicillin/streptomycin, 30 U mL^−1^ of recombinant mouse IL‐2, and 0.05 mM 2‐mercaptoethanol. After incubation for 3 days, activated T cells were further expanded in RPMI medium containing IL‐2 (10 ng mL^−1^) at a density of 0.5–1 × 10^6^ cells mL^−1^.

### Dendritic Cells (DCs) Extraction and Culture

DCs were differentiated from bone marrow cells extracted from the tibias and femurs of C57BL/6 mice. The collected bone marrow cells were incubated using GM‐CSF to differentiate into DCs and gradually mature. DCs were used on days 6th or 7th with a maturation level of ≈ 10% (percentage of CD80^+^CD86^+^ cells in CD11c^+^ cells).

### Construction and Characterization of Glycopolymer‐Engineered T (G‐T) Cells

Activated T cells were plated into 24‐well non‐treated dishes and incubated with Ac_4_ManNAz for 72 h. After washing, T cells were incubated with DBCO‐Cy5 for 30 min. The stained T cells were washed and gathered for FACS analysis. Azido‐labeled T (N_3_‐T) cells were incubated with DBCO‐pMAG/DBCO‐p(MAG‐*co*‐FITC) for 30 min on ice to obtain glycopolymer‐engineered T cells. G‐T cells were washed, collected for flow cytometry analysis, and subsequently observed with a laser scanning confocal microscope.

### In Vitro Cytotoxicity Analysis

To confirm the cytotoxicity in bioorthogonal labeling of T cells, the cell viability of T cells was monitored using CCK‐8 after treatment with Ac_4_ManNAz or DBCO‐pMAG, respectively. To optimize treatment concentrations of Ac_4_ManNAz forT cells, T cells were incubated with various concentrations (0, 12.5, 25, 50, 75, and 100 µm) of Ac_4_ManNAz for 3 days on the one hand. N_3_‐T cells were then washed once with PBS and were transferred into 96‐well plates (2×10^5^ cells well^−1^). On the other hand, T cells were incubated with 50 µM concentrations of Ac_4_ManNAz for 3 days at 37 °C. Then N_3_‐T cells were washed twice with PBS, incubated with various concentrations of DBCO‐pMAG for 30 min and then transferred into 96‐well plates (1×10^5^ cells well^−1^). A mixture of 20 µL of CCK‐8 solution and 200 µL medium was added to each well, followed by an incubation of 2 h at 37 °C. To calculate cell viability, the absorbance of each well was measured using a multifunctional microplate reader at 450 nm.

### Cell Image Tracking and Analysis

Cell image tracking was performed by the Nikon Eclipse Ti Imaging System. The tumor cells were placed into the 24‐well plate 8 h earlier. Half an hour before filming, T cells were added to the plate. Images were acquired sequentially every 1 min for 120 min. The contact between tumor cells and T cells was indicated by manually counting the number of T cells and contact duration from at least eight independent samples.

### Quartz Crystal Microbalance (QCM) Measurements

The QCM chips (5 MHz, Ti, AT‐cut, 14 mm in diameter) were washed with DIW and ethanol iteratively. Both sides of the chips were exposed to UV for 30 min and cleaned with ethanol and DIW respectively, and then placed into QCM chamber. Phosphate buffered saline (PBS) was injected into the system at a flow rate of 10 µL min^−1^. Until the baseline was stable, the freshly prepared glycopolymer solution (2 mg mL^−1^) was introduced for 1 h. After that, the chips were washed with PBS to remove the loosely connected sugar polymers to obtain a new stable baseline.

### Construction of GLUT1‐ B16‐OVA Cells

The gRNA sequences were gRNA1 (5’‐GGATGGGCTCTCCGTAGCGGTGG‐3’), gRNA2 (5’‐CGTGGCCATCTTCTCTGTCGGGG‐3’), and gRNA3 (5’‐AGACCAAAGCGTGGTGAGTGTGG‐3’). 1 µg of each gRNA plasmid was transfected into B16‐OVA cells with the Lipofectamine 3000 Reagent according to the manufacturer's instruction. 48 h after transfection, B16‐OVA cells were treated with 2 µg mL^−1^ puromycin for 48 h. The knock‐out effect of B16‐OVA cells was confirmed by Western Blot.

### Western Blot

Cells were lysed in 100 µL of RIPA buffer supplemented with protease inhibitor cocktail per tube. The suspensions were placed on ice and subjected to ultrasonication at 80 W for three cycles of 6 s pulses with 10 s intervals. Lysates were clarified by centrifugation at 12,000 rpm for 3 min at 4 °C, and the supernatants were collected. Protein samples were mixed with 4× reducing loading buffer, heated at 100 °C for 10 min, cooled to room temperature, and briefly centrifuged prior to electrophoresis. Equal volumes of denatured protein samples were loaded for SDS‐PAGE.

### LDH Release Assay

B16‐OVA cells were seeded onto 48‐well plate (1 × 10^4^ cells well^−1^) in RPMI 1640 medium. After 8 h, activated T cells, N_3_‐T cells or G‐T cells from OT‐1 mice were added to B16‐OVA cells at different effector‐to‐target (E/T) ratios. After co‐incubation for 4 h, supernatants were used for LDH cytotoxicity assay using a LDH cytotoxicity assay kit according to the manufacturer's instruction.

### B16‐OVA Tumor Immunotherapy with G‐T Cells

Tumor‐bearing C57BL/6 mice were randomly divided into three groups (untreated group, T cells group or G‐T cells group). Then mice were injected intravenously PBS, T cells, G‐T cells (100 µL, 1 × 10^7^ cells per mouse) for the treatment on the 6th, 11th, and 16th days after tumor inoculation. The bodyweight of the mice, survival rate, and tumor volume were recorded every 2 days during the period of treatment. Mice were euthanized when tumor volumes exceeded 1000 mm^3^ in accordance with humane endpoint criteria. Single cell suspensions were prepared from half of dissected tumor tissues of tumor‐bearing mice. The enrichment of T cells and maturation of DCs in tumor tissues were analyzed by flow cytometry. For cytokine assays in vivo, the other half of the tumor tissues were weighed, sliced, digested, filtered, and centrifuged. According to the instructions, the concentration of TNF‐α, IFN‐γ, Granzyme B and IL‐12 p70 was detected by the corresponding ELISA kit.

### CT‐26 Tumor Immunotherapy Combined with G‐T Cells and PD‐1 Antibody

CT‐26 cell membrane was prepared by mechanical disruption and centrifuged at 14,800 rpm. for 20 min to remove the cell nuclei and cytoplasm. The obtained membrane was selected as the antigen, and CpG as the activator to construct antigen‐presenting DCs.^[^ ^39^ ^]^ The obtained membranes were quantified with a BCA assay kit and the concentration of the final medium was 5 µg mL^−1^. The CpG concentration was 1 µg mL^−1^. Then T cells and DCs were incubated in round bottom 96‐well plates at a DC: T cell ratio of 1:10 for 72 h to obtain CT‐26‐specific T cells. For in vivo studies, CT‐26 cells (50 µL, 2 × 10^5^ cells) were subcutaneously injected into the backs of female BALB/c mice to establish the primary tumor. The mice were randomly divided into 4 groups (untreated group; aPD‐1 group; G‐T group; G‐T@aPD‐1 group) when the tumor size reached ≈50 mm^3^. G‐T cells were intravenously injected on the 6th and 12th days after tumor inoculation, while PD‐1 antibody was administered on the 9th and 15th days at a dose of 20 µg per mouse.

### Statistical Analysis

Statistical analyses were performed using GraphPad Prism software (Version 9.02). Except where indicated differently, all values and error bars are mean ± SD. Detailed statistical methods are described in the figure legends. n.s. > 0.05, *p < 0.05, **p < 0.01, ***p < 0.001, ****p < 0.0001.

## Conflict of Interest

The authors declare no conflict of interest.

## Supporting information



Supporting Information

## Data Availability

The data that support the findings of this study are available from the corresponding author upon reasonable request.
