# Supplementary material for: T Cell Glycoengineering to Modulate Immune‐Tumor Crosstalk: A Universal Non‐Genetic Strategy for Enhanced Tumor Immunotherapy
Source: Adv Sci (Weinh). 2025 Dec 8;13(8):e05387. doi: 10.1002/advs.202505387 (PMC12884725; doi:10.1002/advs.202505387)
Supplement: Supplementary file 1 — Supporting Information [file ADVS-13-e05387-s001.docx]

Supporting Information

**T Cell Glycoengineering to Modulate Immune-Tumor Crosstalk: A Universal Non-Genetic Strategy for Enhanced Tumor Immunotherapy**

Lihua Yao, He Yang^*^, Fangjian Shan, Xiaomeng Niu, Yichen Wang, Hengyuan Zhang, Sujian Wang, Gaojian Chen^*^ and Hong Chen^*^

Lihua Yao, He Yang, Fangjian Shan, Xiaomeng Niu, Yichen Wang, Hengyuan Zhang, Sujian Wang, Gaojian Chen

State Key Laboratory of Bioinspired Interfacial Materials Science, State and Local Joint Engineering Laboratory for Novel Functional Polymeric Materials, College of Chemistry, Chemical Engineering and Materials Science, Soochow University, Suzhou, 215123, P. R. China

Hong Chen

State Key Laboratory of Bioinspired Interfacial Materials Science, State and Local Joint Engineering Laboratory for Novel Functional Polymeric Materials, College of Chemistry, Chemical Engineering and Materials Science, Soochow University, Suzhou, 215123, P. R. China

Jiangsu Biosurf Biotech Co., Ltd., Suzhou, 215123, P. R. China

E-mail: chenh@suda.edu.cn

**Table S1**. Reaction conditions of pMAG and p(MAG-*co*-FITC).

| **Glycopolymers** | **MAG:CPADB:AIBN:FluMA** | **Solvent** |
| --- | --- | --- |
| **pMAG** | **50：1：0.6：0** | **DMSO** |
| **p(MAG-*co*-FITC)** | **50：1：0.6：2** |  |


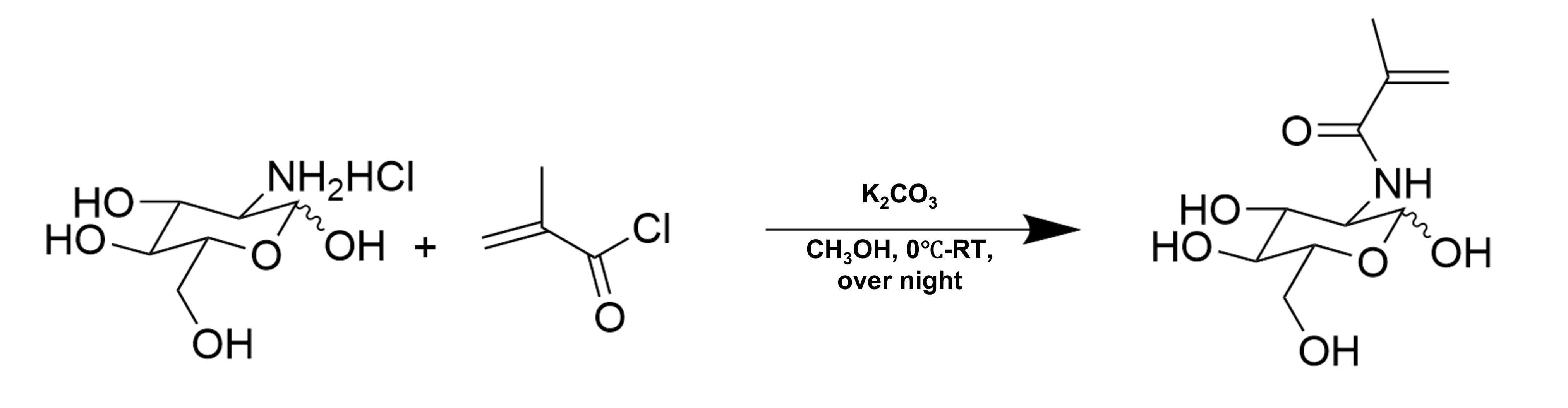


**Figure S1.** Synthetic route of MAG.


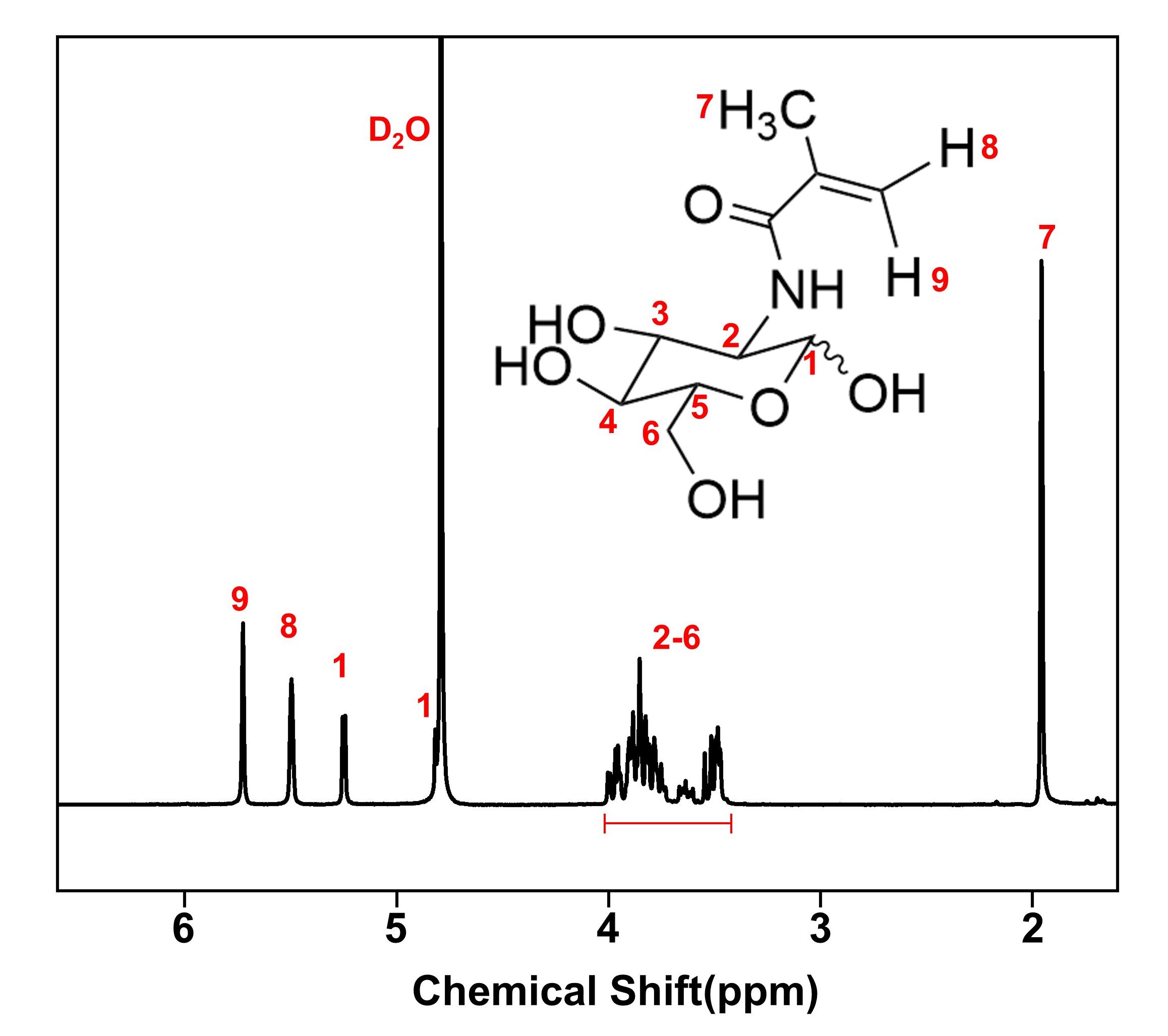


**Figure S2.** ^1^H NMR spectrum of MAG in D_2_O. ^1^H NMR (300 MHz, Deuterium Oxide, δ) 5.79-5.67 (m, 1H), 5.54-5.42 (m, 1H, CH_2_), 5.25 (d, *J* = 3.5 Hz, 0.6H, pyranose H), 4.82 (s, 0.4H, pyranose H), 4.04-3.39 (m, 6H, pyranose H), 2.01-1.82 (m, 3H, CH_3_).


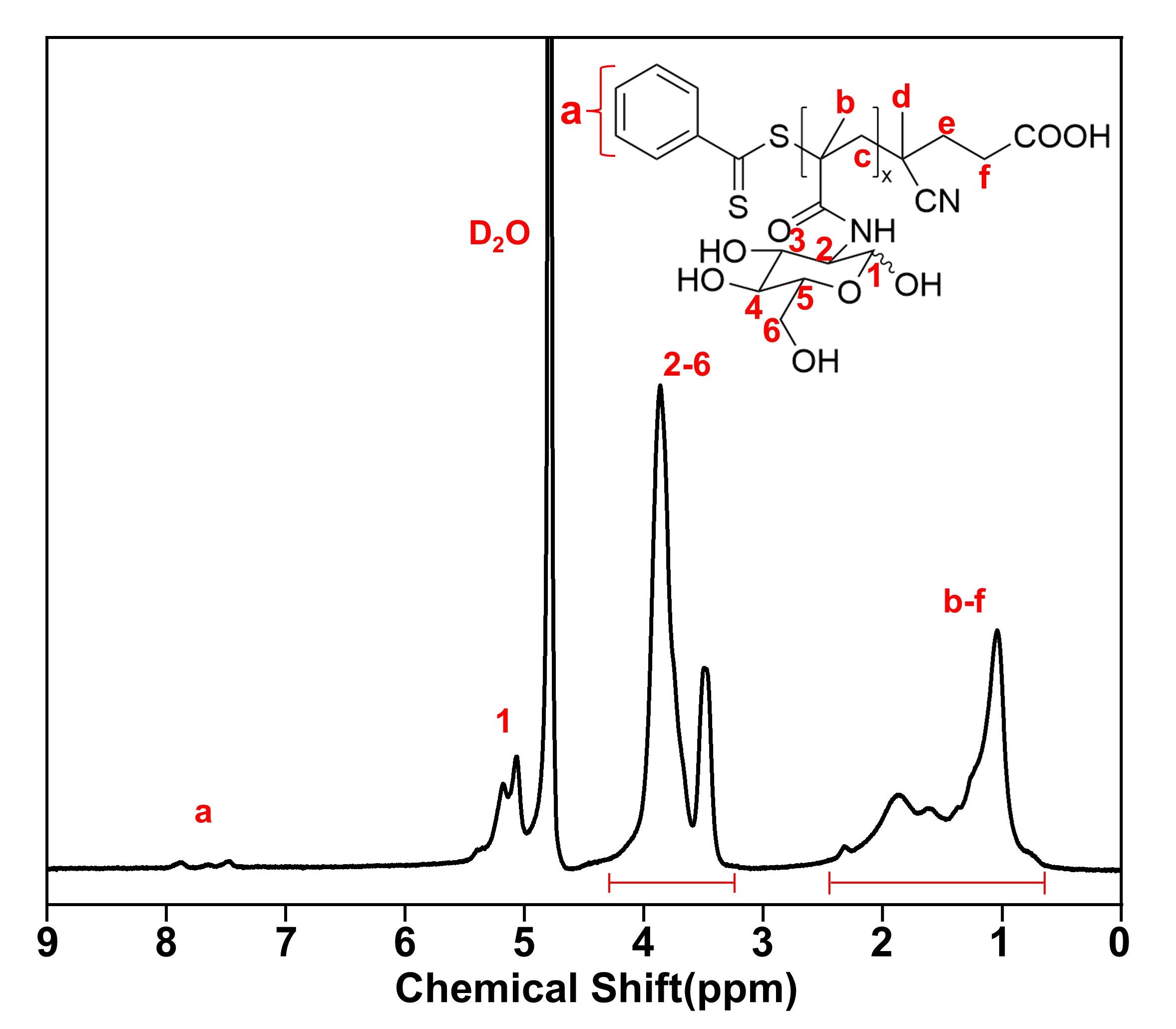


**Figure S3.** ^1^H NMR spectrum of pMAG in D_2_O.


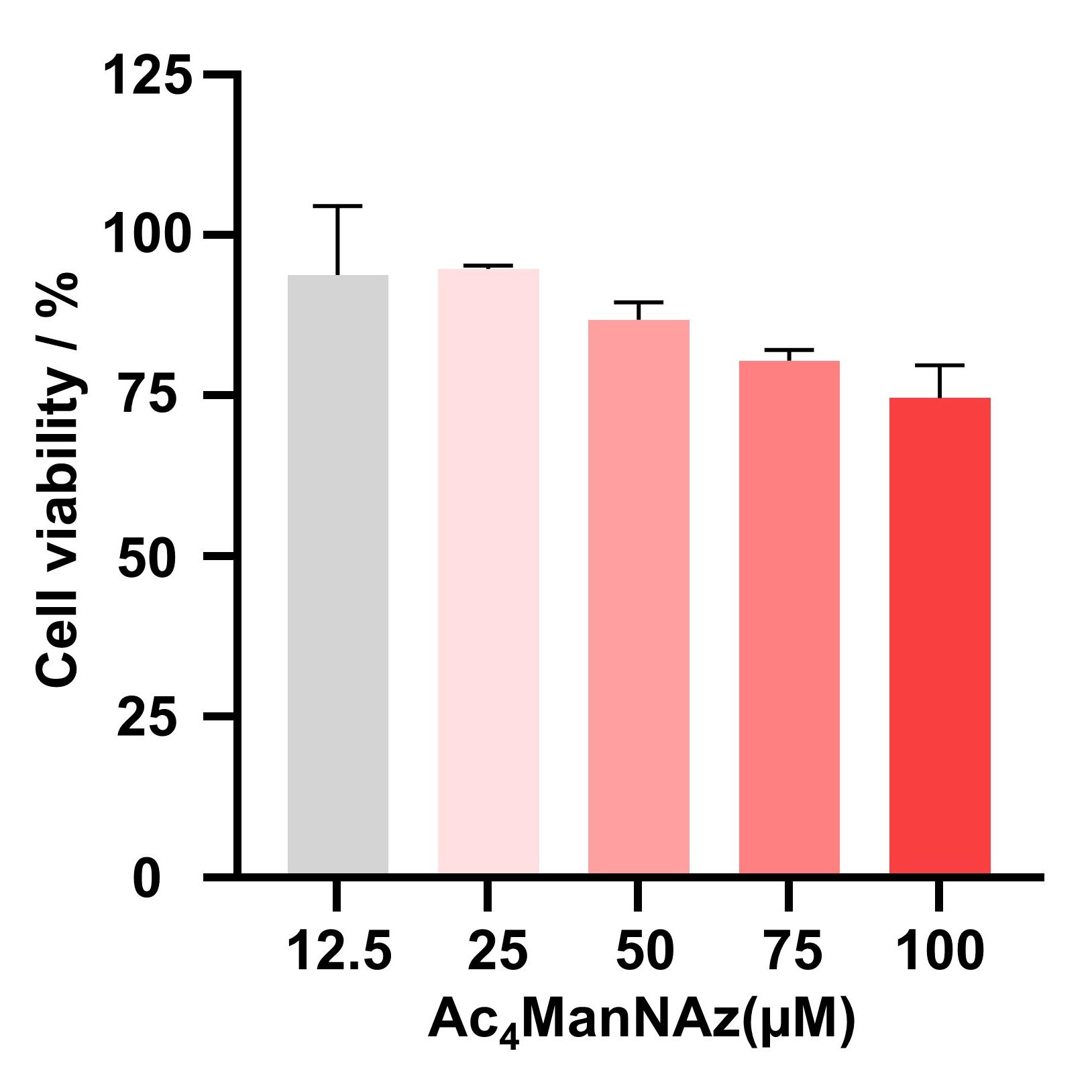


**Figure S4.** Effects of Ac_4_ManNAz with different concentrations on cell viability.


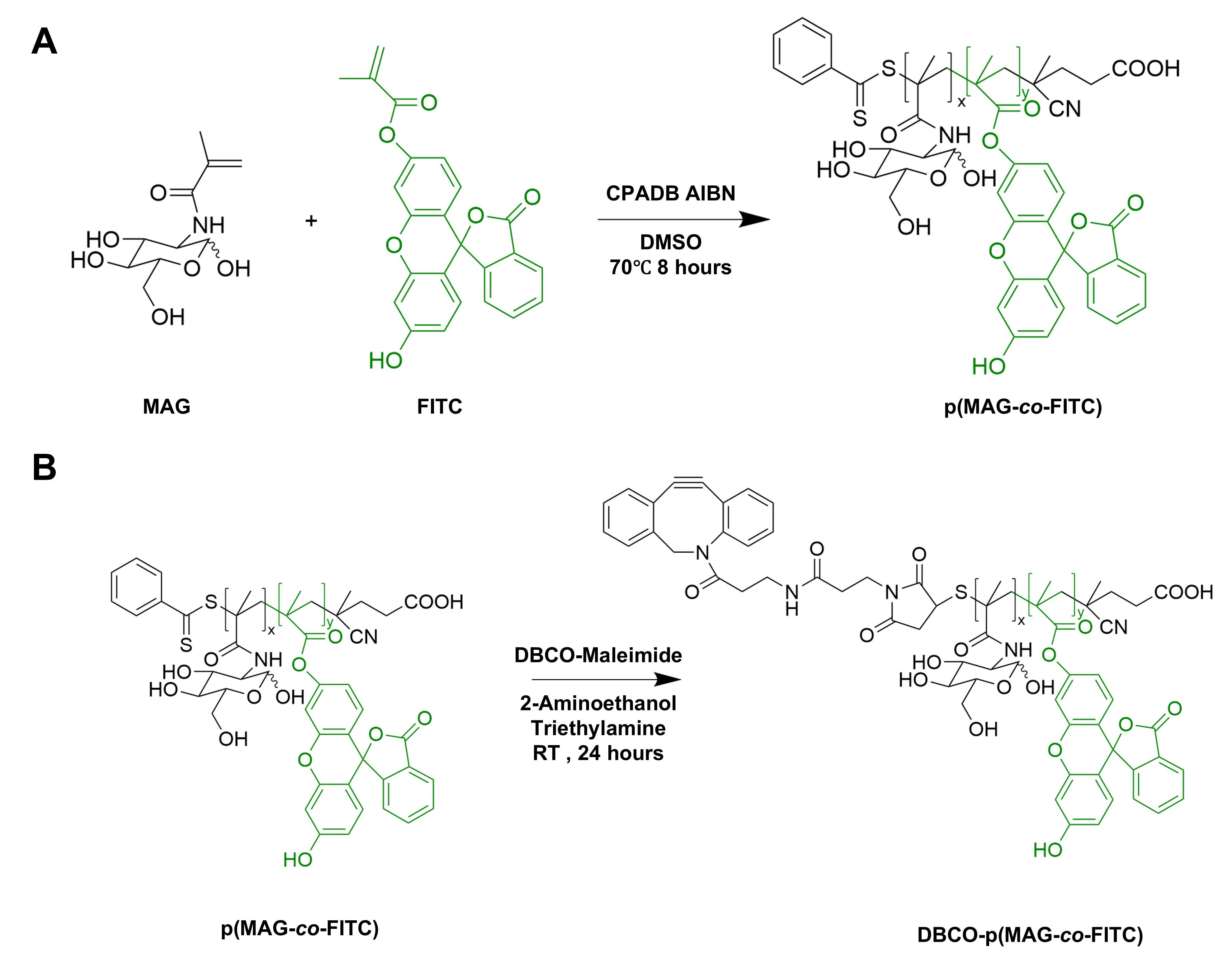


**Figure S5.** Synthetic route of (A) p(MAG-*co*-FITC) and (B) DBCO-p(MAG-*co*-FITC).


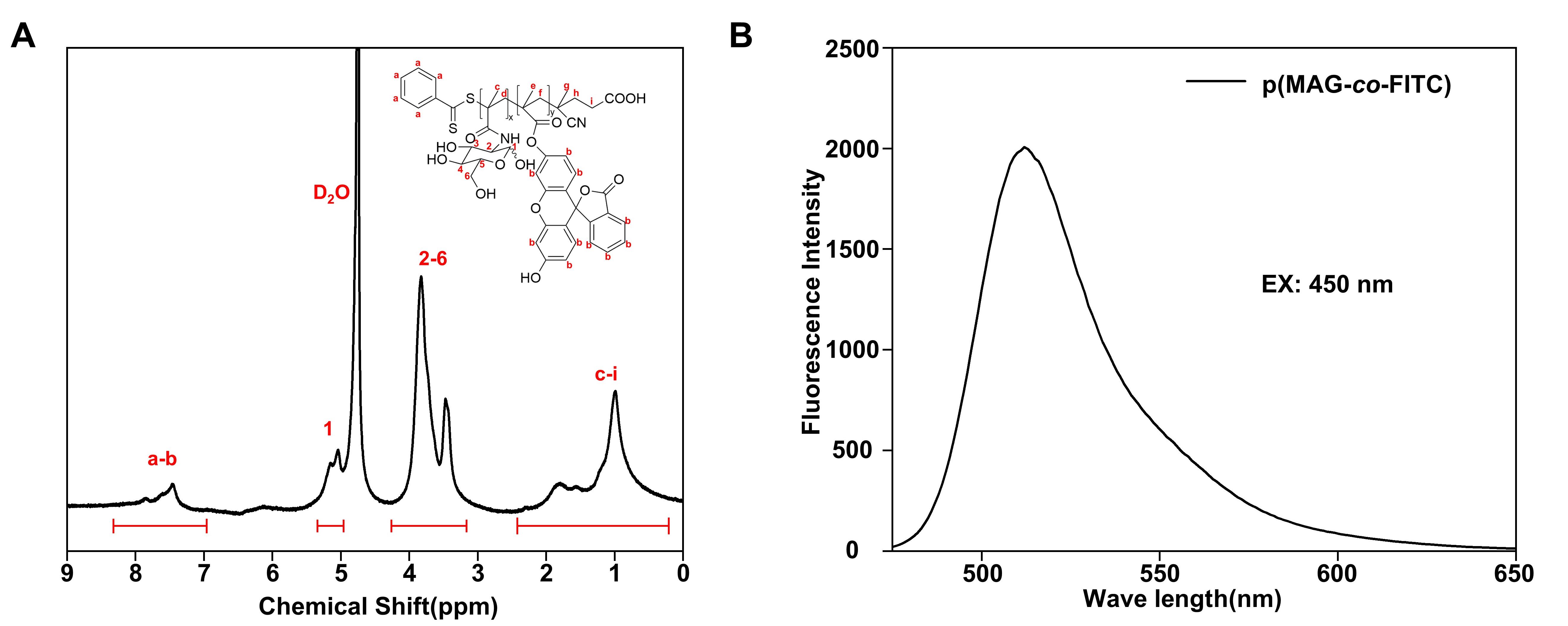


**Figure S6.** (A) ^1^H NMR spectrum of p(MAG-*co*-FITC) in D_2_O. (B) Fluorescence spectrum of p(MAG-*co*-FITC).


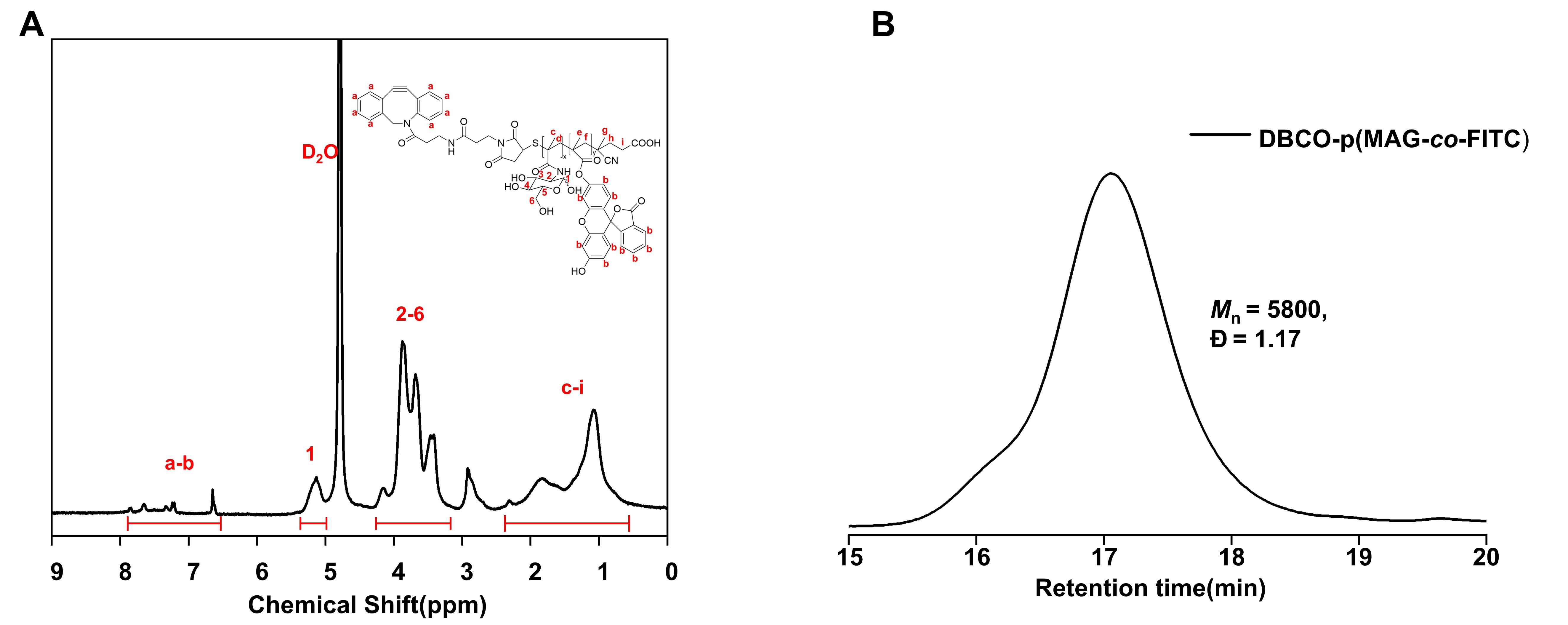


**Figure S7.** (A) ^1^H NMR spectrum of DBCO-p(MAG-*co*-FITC) in D_2_O. (B) SEC traces of DBCO-p(MAG-*co*-FITC).


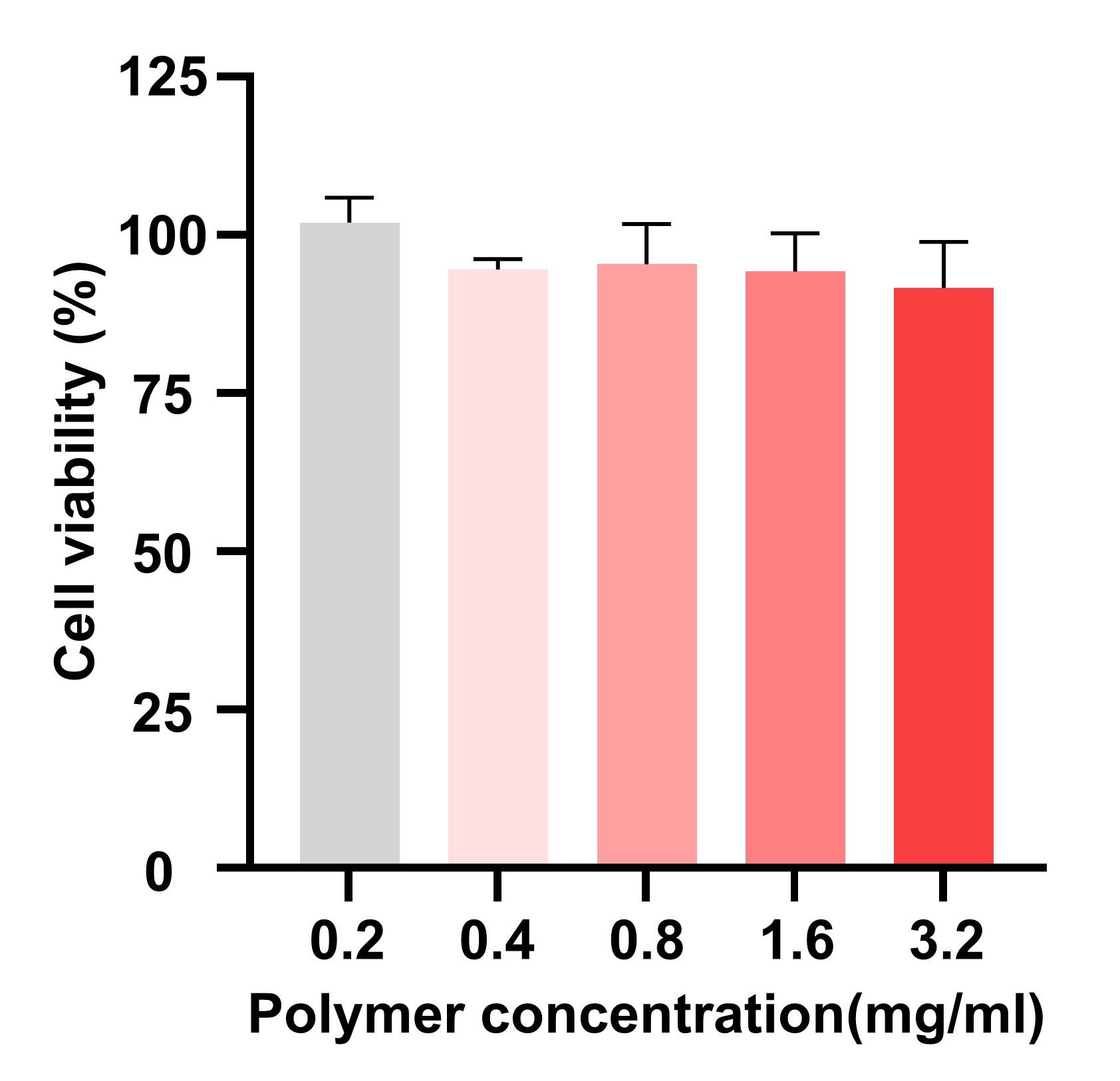


**Figure S8.** Effects of DBCO-pMAG with different concentrations on cell viability.


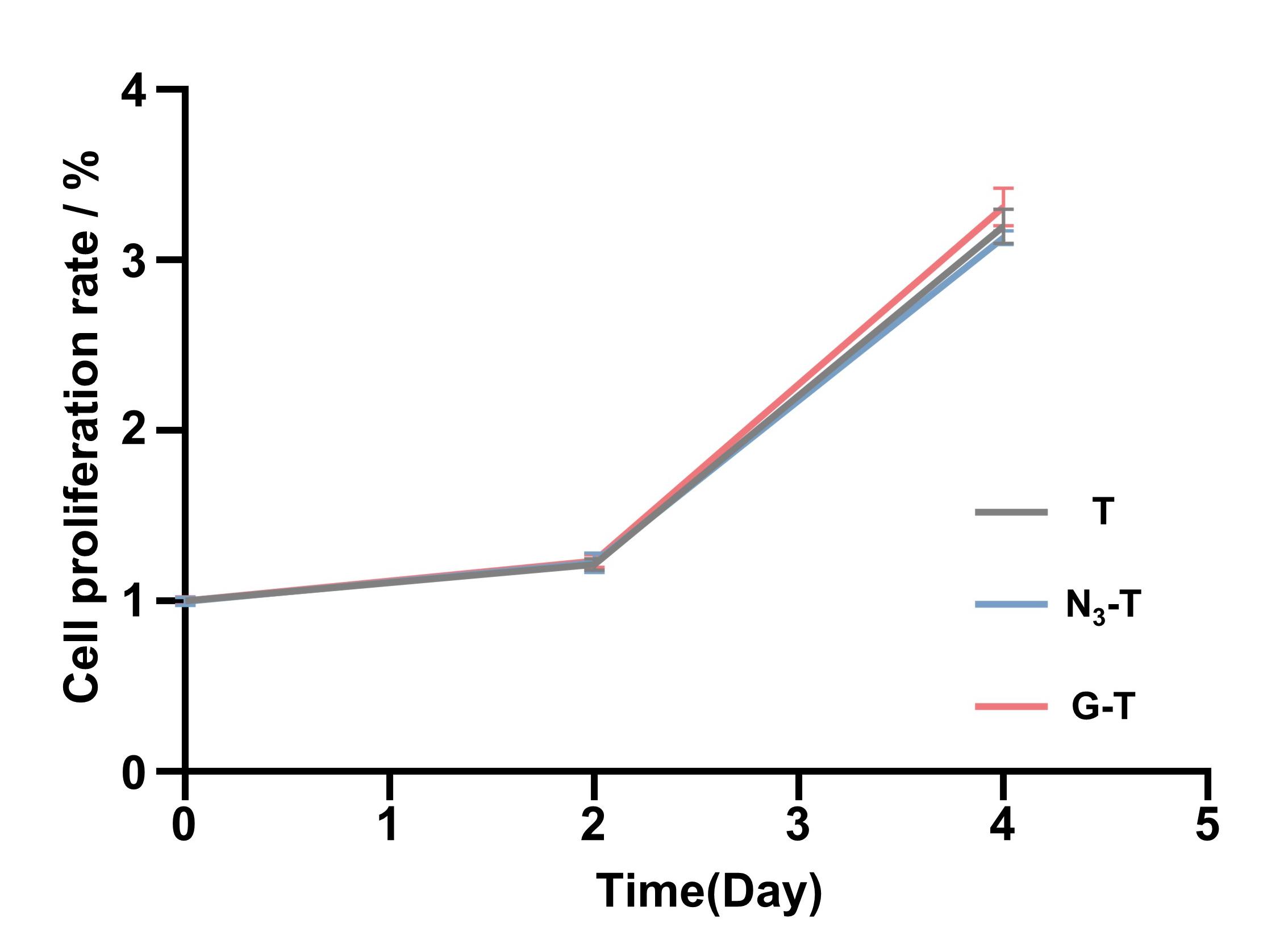


**Figure S9.** Cell count in T cells over time.


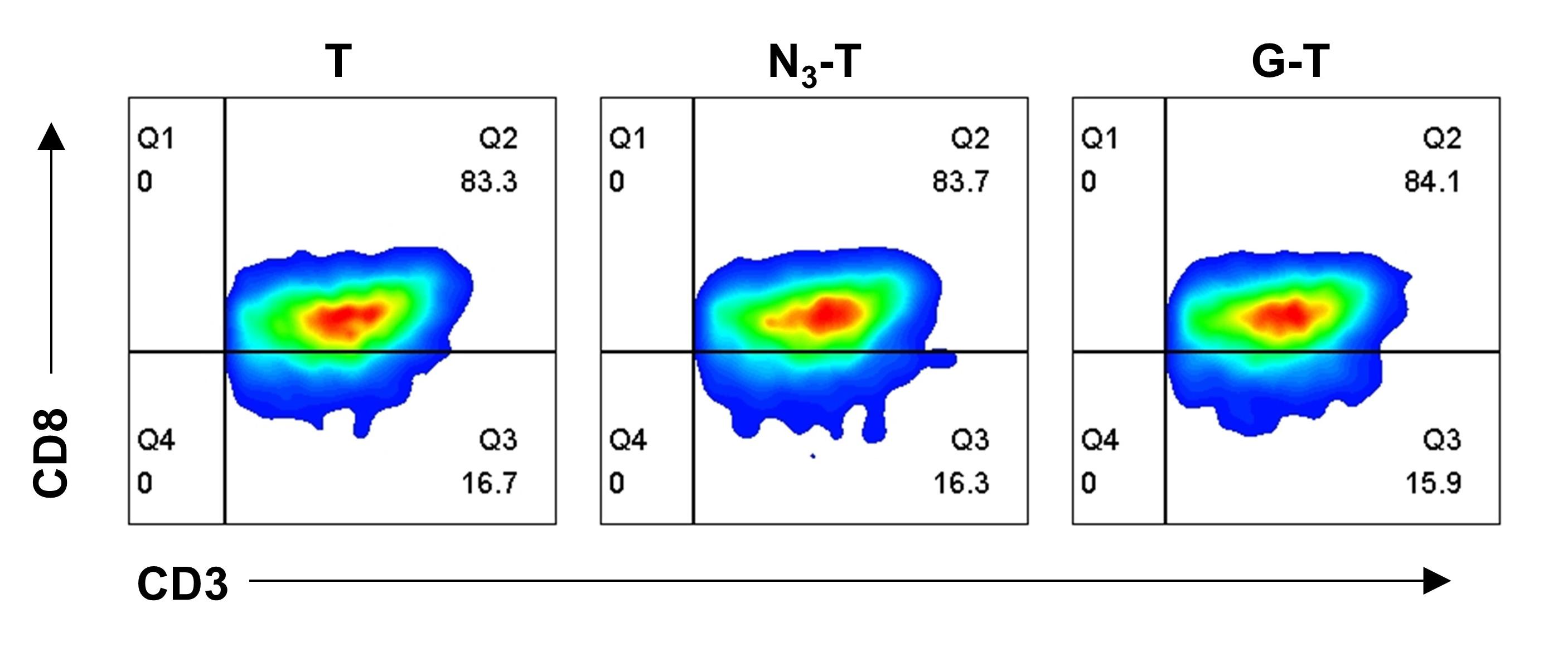


**Figure S10.** Flow cytometry analysis of CD3^+^ and CD8^+^ T cells (gated on CD3^+^ T cells) after different treatment.


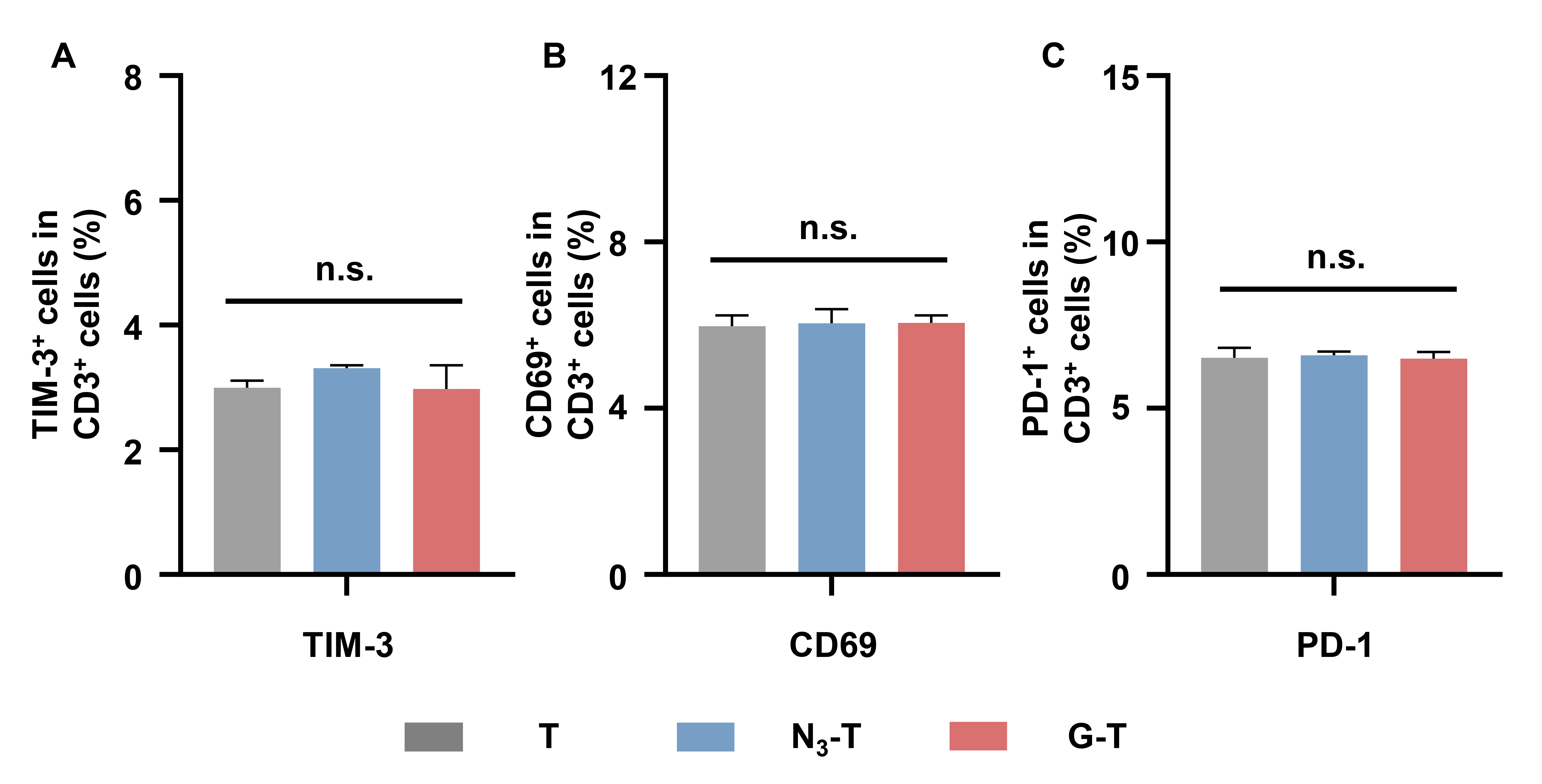


**Figure S11.** Flow cytometry analysis of (A) TIM-3^+^ cells, (B) CD69^+^ cells and (C) PD-1^+^ cells (gated on CD3^+^ T cells) after different treatment. Data are presented as mean ± SD (n = 3). Statistical analysis was performed using Tukey’s multiple comparison test and, n.s.>0.05.


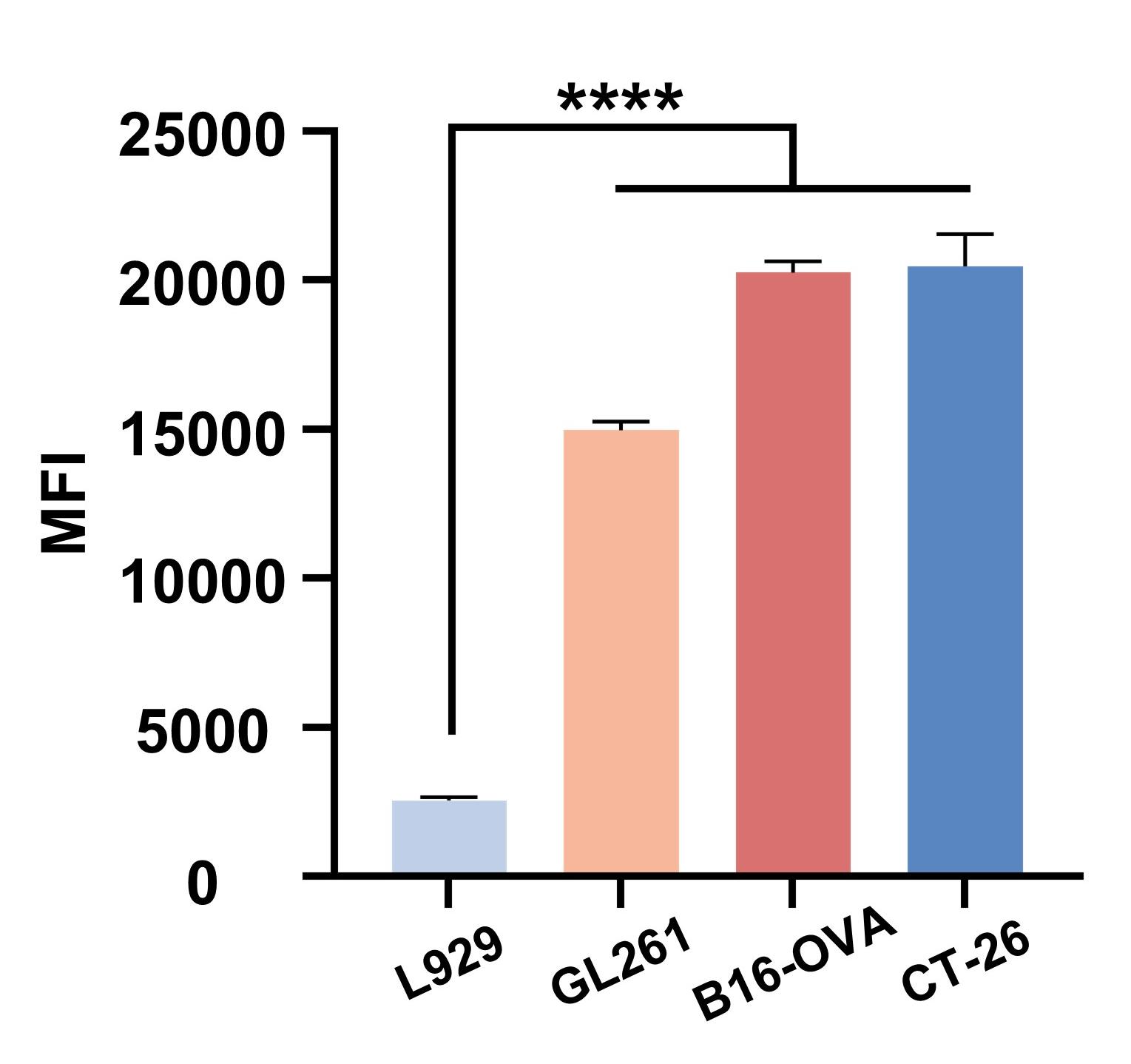


**Figure S12.** Flow cytometry statistics of expressed GLUT1 expression (Mean Fluorescence Intensity = Experimental group - Control group）on L929, GL261, B16-OVA and CT-26 cells. The error bars represent mean ± SD (n = 3). ****p < 0.0001.


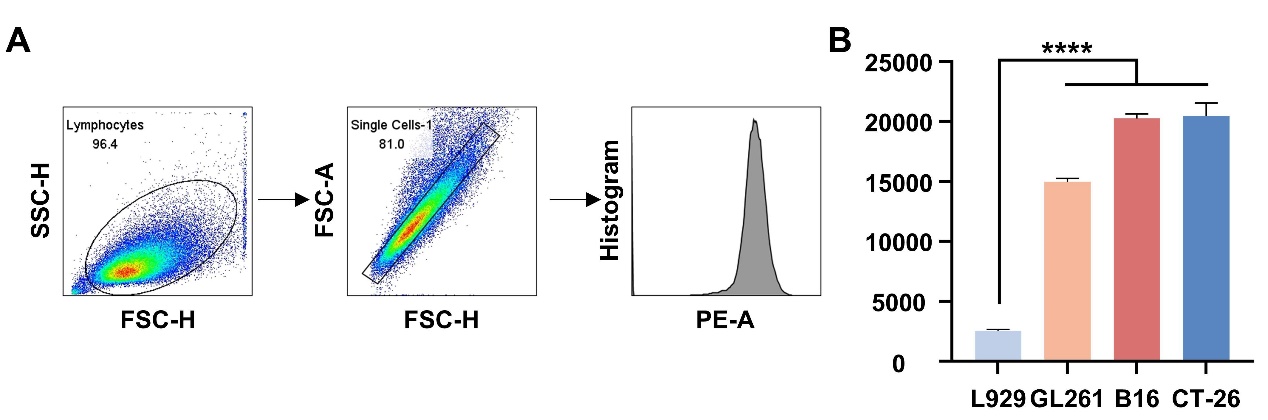


**Figure S13.** Gating strategies for flow cytometry analysis of GLUT1 expression on cells.


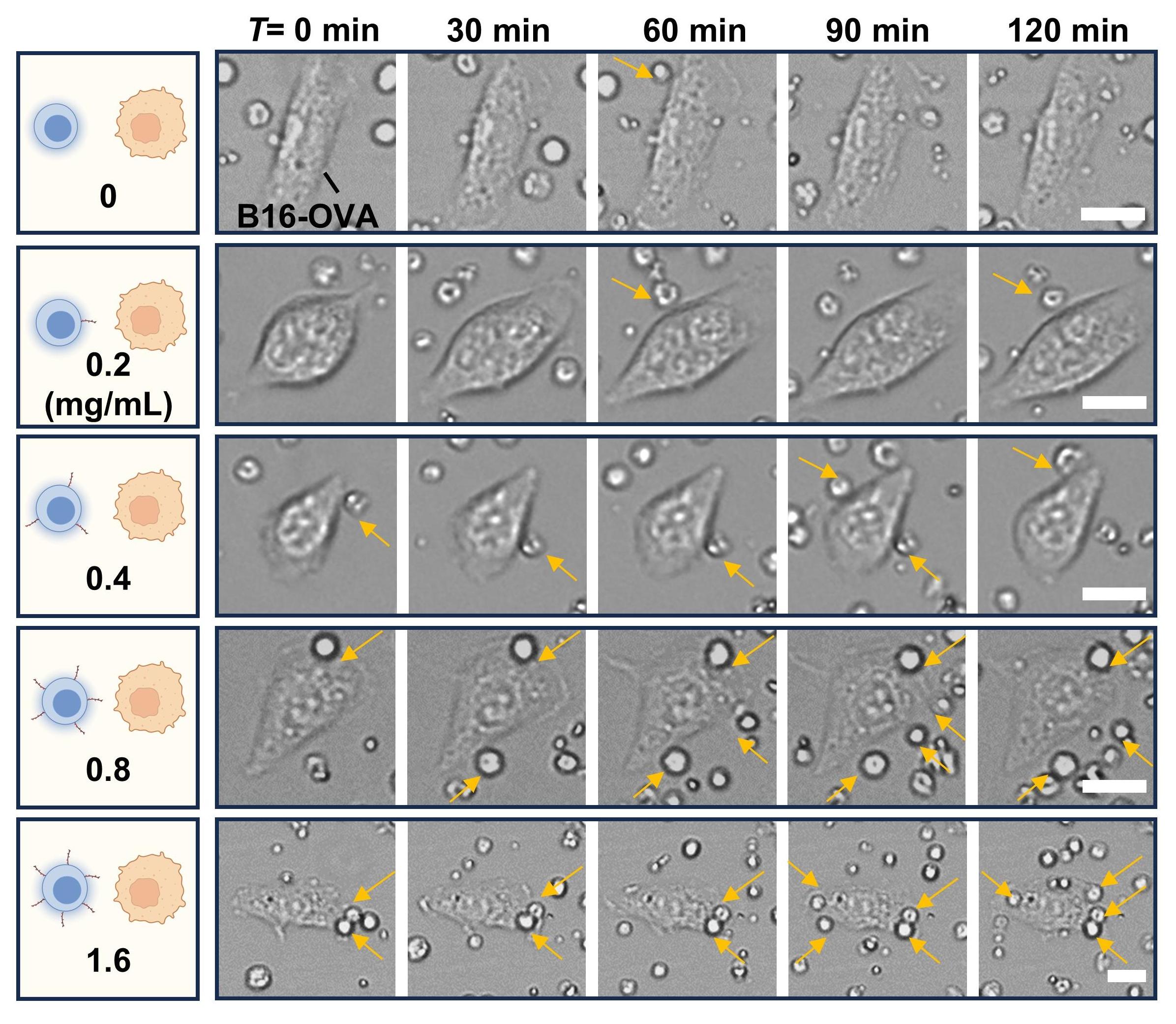


**Figure S14.** Image tracking of T cells and B16 cells migration over time at 10:1 E/T ratio. Scale bar = 10 µm.


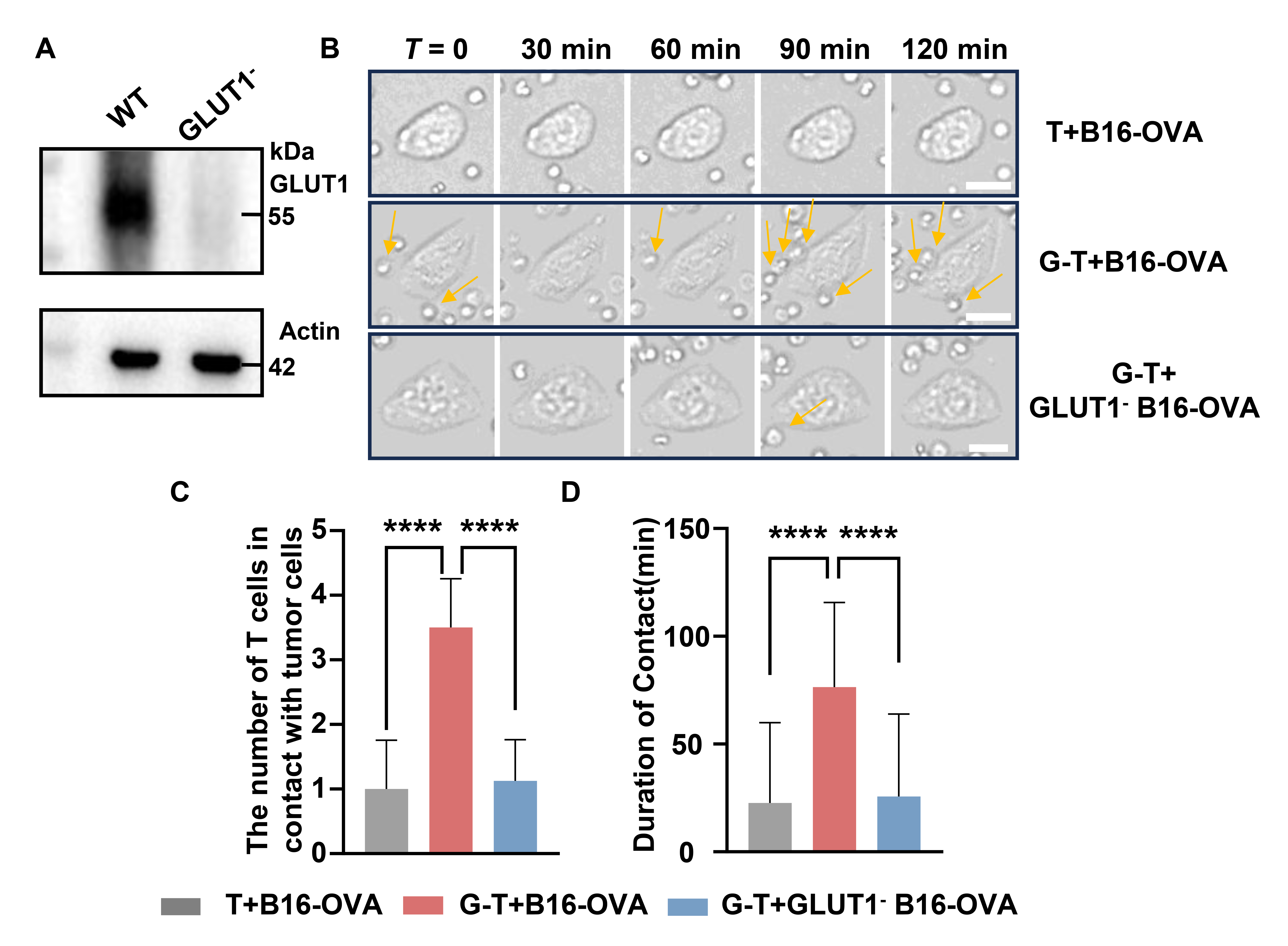


**Figure S15.** Investigation of contact behavior between G-T cells and GLUT1^-^ B16-OVA cells. (A) GLUT1 expression in the indicated groups was detected by Western blotting. (B) Image tracking of G-T cells and GLUT1^-^ B16-OVA cells migration over time at 10:1 E/T ratio. Scale bar = 10 µm. (C) The number of T cells in contact with B16-OVA cells, with B16-OVA cells number (n = 8). (D) Duration of contact between T cell and B16-OVA cells, with T cell number (n = 25). The error bars represent mean ± SD. Statistical analysis was performed using Tukey’s multiple comparison test and, ****p < 0.0001.


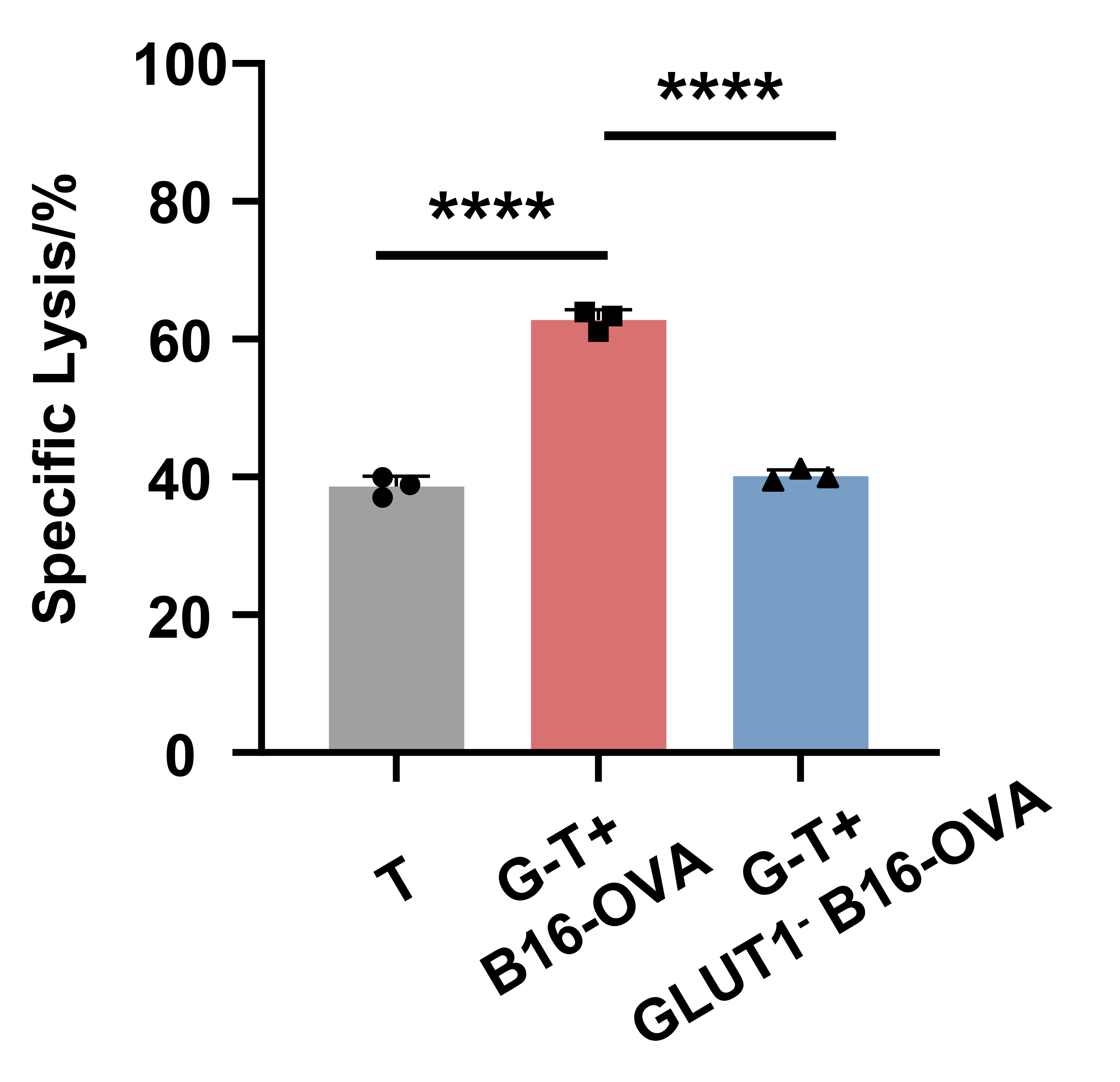


**Figure S16.** The lysis efficacies of GLUT1^-^ B16-OVA cells by T cells at the E/T ratios of 10:1. The error bars represent mean ± SD (n = 3). Statistical analysis was performed using Tukey’s multiple comparison test and, ****p < 0.0001.


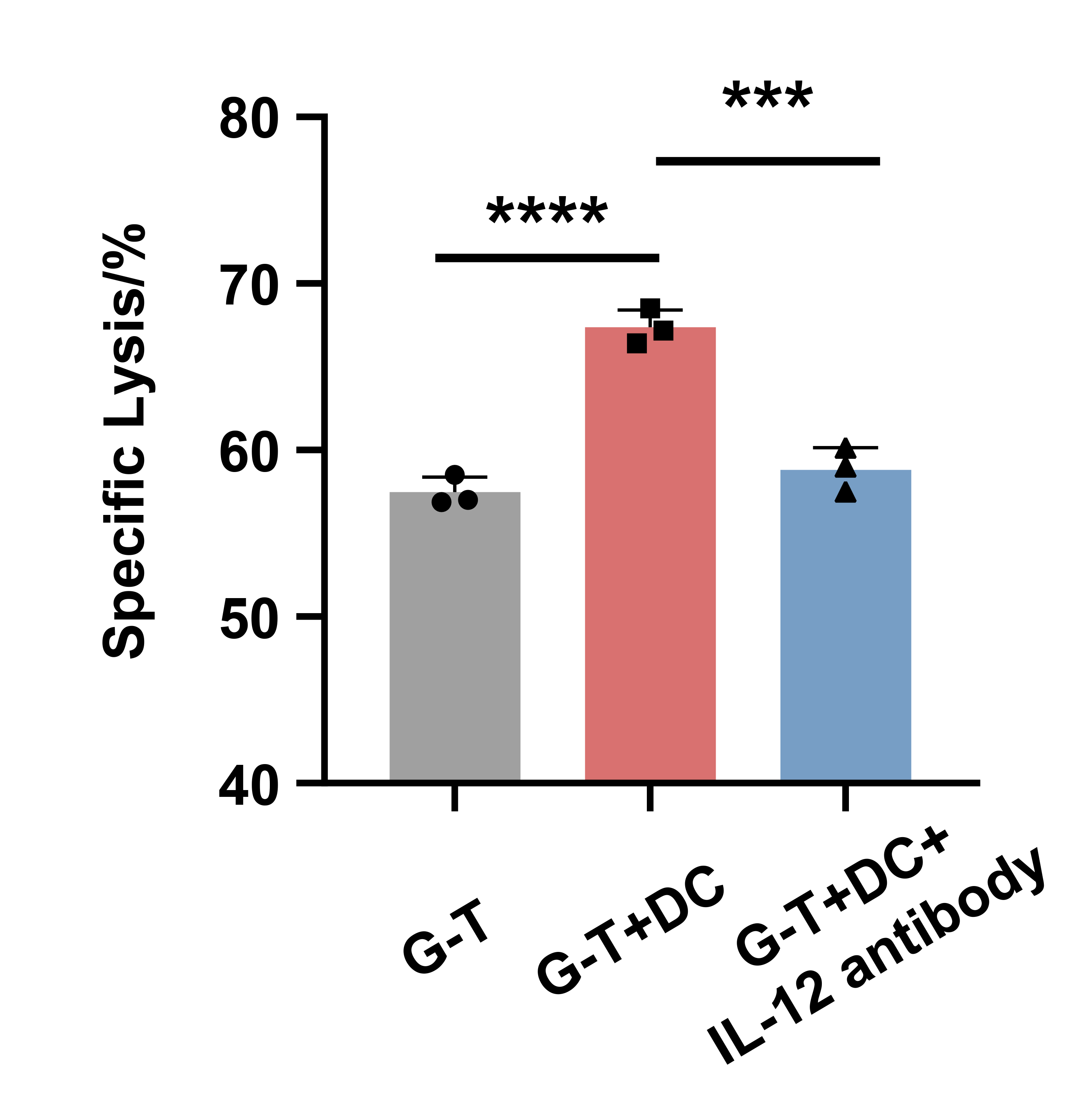


**Figure S17.** The lysis efficacies of tumor cells after incubation with G-T cells and DCs. The error bars represent mean ± SD (n = 3). Statistical analysis was performed using Tukey’s multiple comparison test and, ***p < 0.001, ****p < 0.0001.


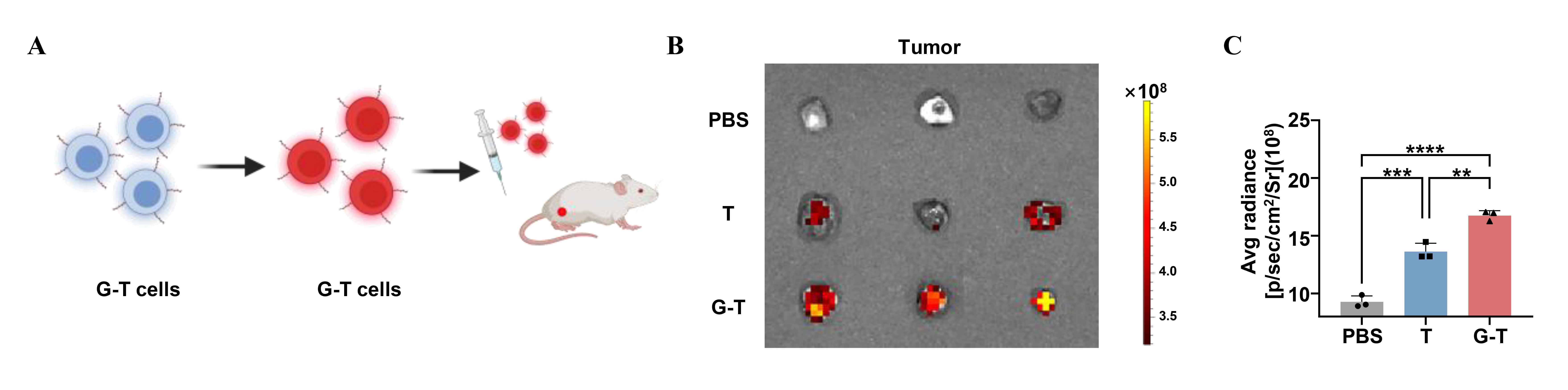


**Figure S18.** (A) Diagram of DiR labeled G-T cells injected intravenously into C57BL/6 tumor-bearing mice. (B) Representative *ex vivo* fluorescence images of tumors at 24 hours after G-T injection (n = 3). (C) Quantitative of T cells fluorescence signal. The error bars represent mean ± standard deviations (n = 3).


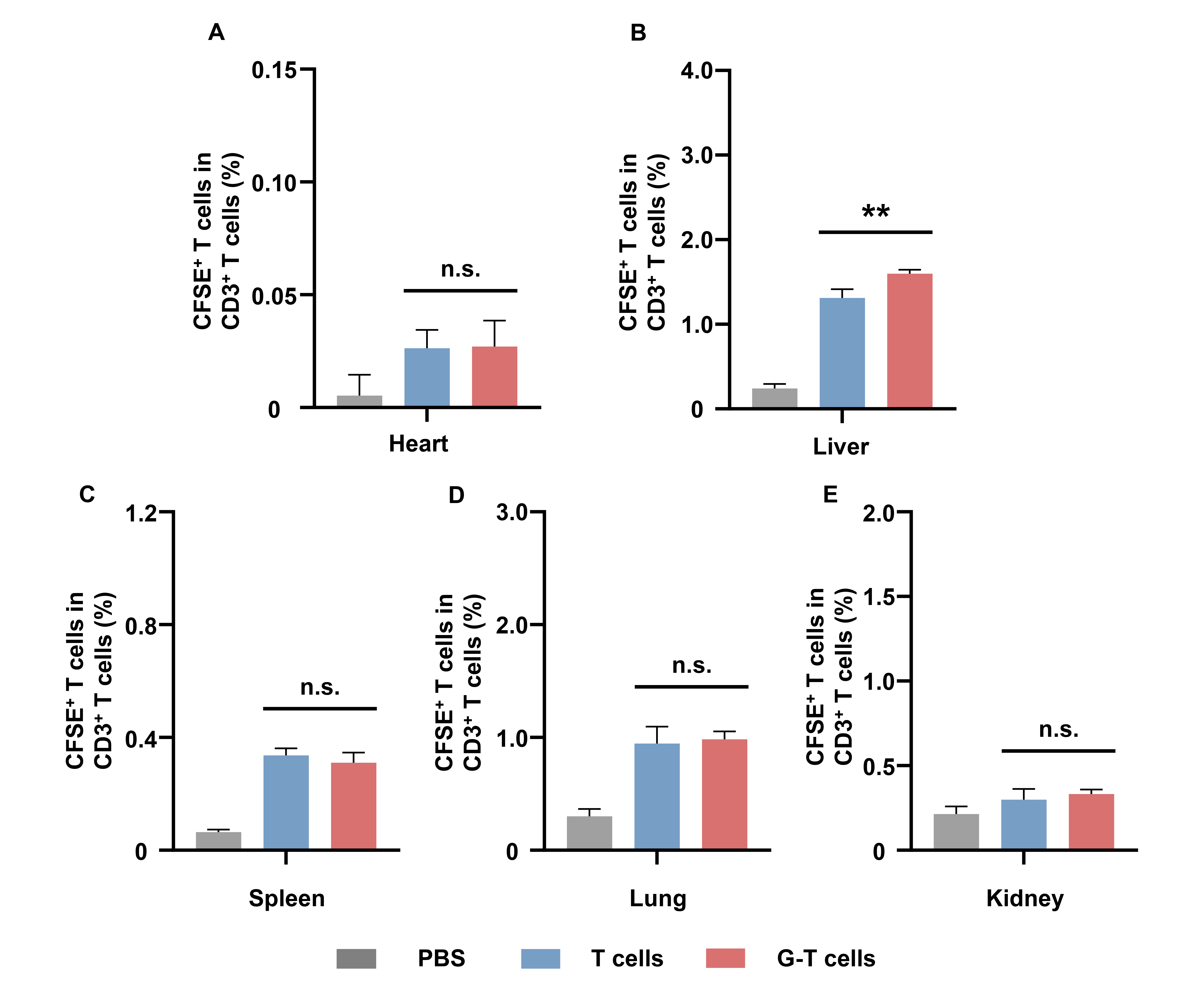


**Figure S19.** Biodistribution of transferred T cells in major organs including (A) heart, (B) liver, (C) spleen, (D) lung, and (E) kidneys. Data are presented as mean ± SD (n = 3). Statistical analysis was performed using Tukey’s multiple comparison test and, n.s.>0.05, **p<0.01.


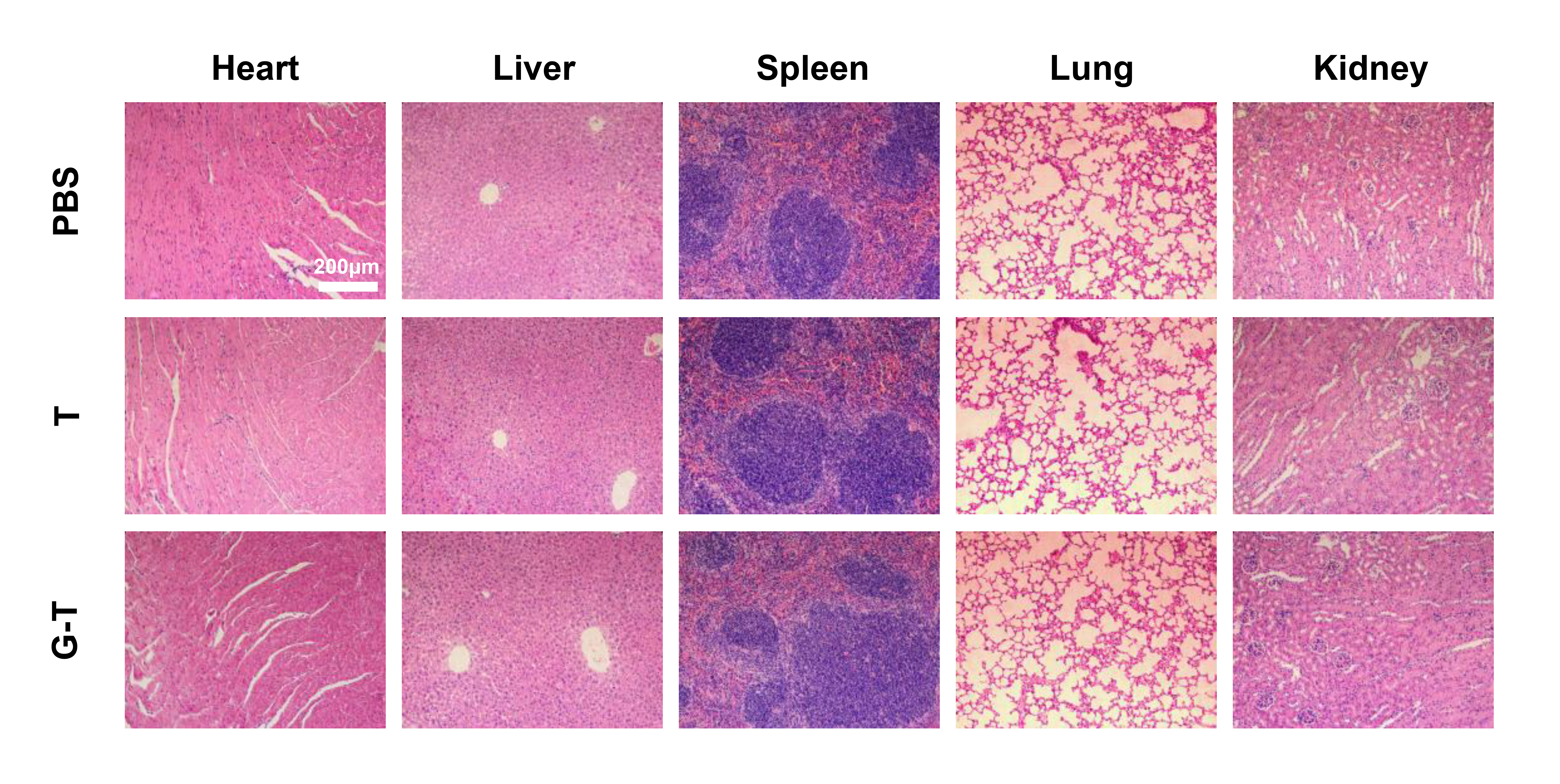


**Figure S20.** Hematoxylin and eosin (HE) staining analysis of heart, liver, spleen, lung and kidney tissue in different treatment groups. Scale bar = 200 μm.


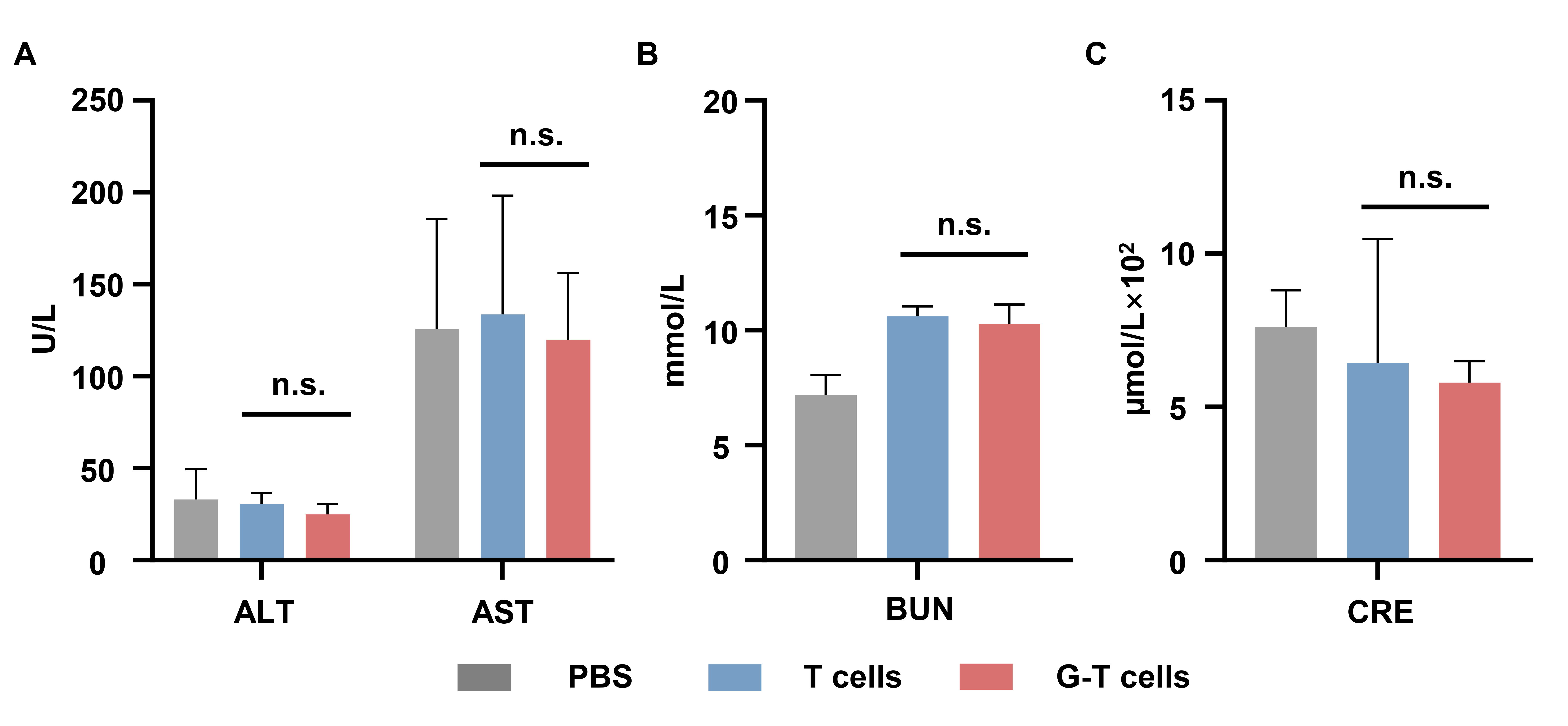


**Figure S21.** Quantitative analysis of indicators for liver and kidney function. (A) alanine aminotransferase (ALT) and aspartate aminotransferase (AST); (B) blood urea nitrogen (BUN); (C) creatinine (CRE) were evaluated of different treatment groups. Data are presented as mean ± SD (n = 3). Statistical analysis was performed using Tukey’s multiple comparison test and, n.s.>0.05.


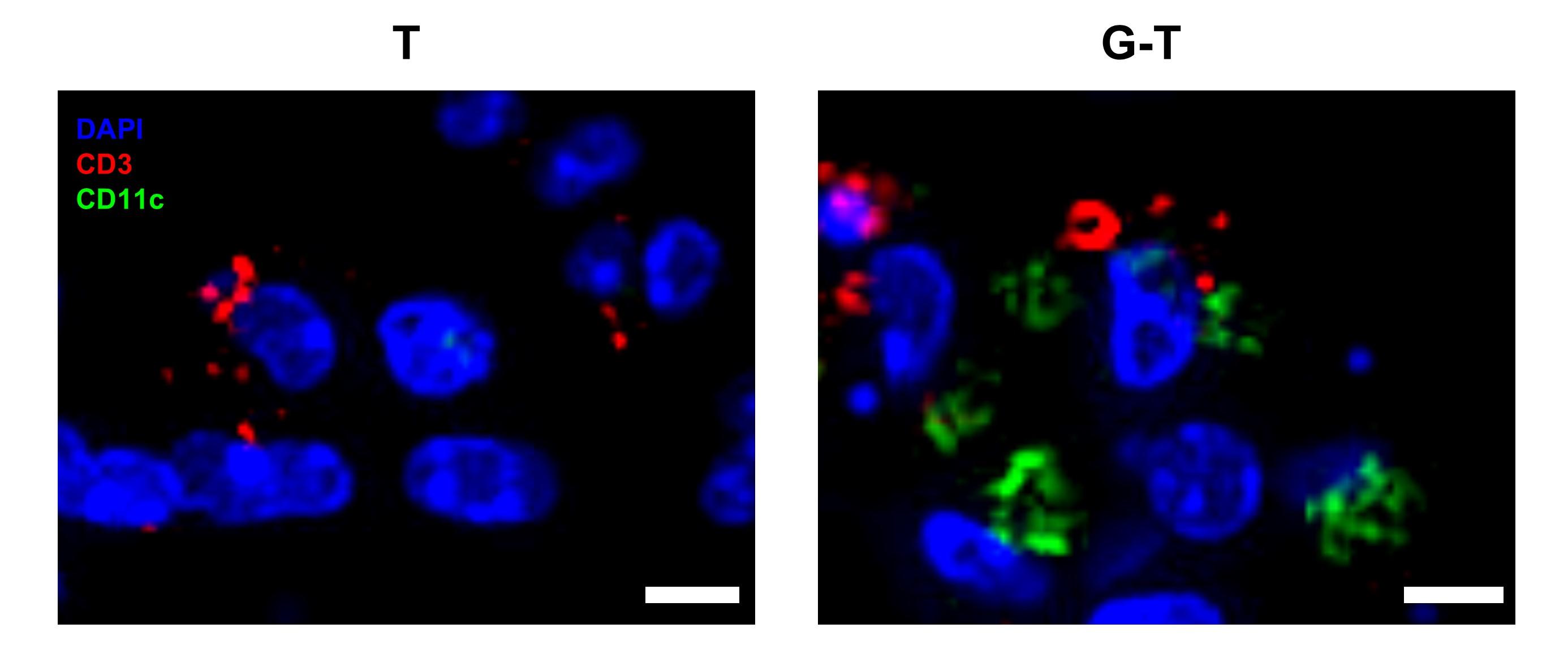


**Figure S22.** CLSM image of CD3^+^ T cells and CD11c^+^ DCs. Scale bar = 5 µm.


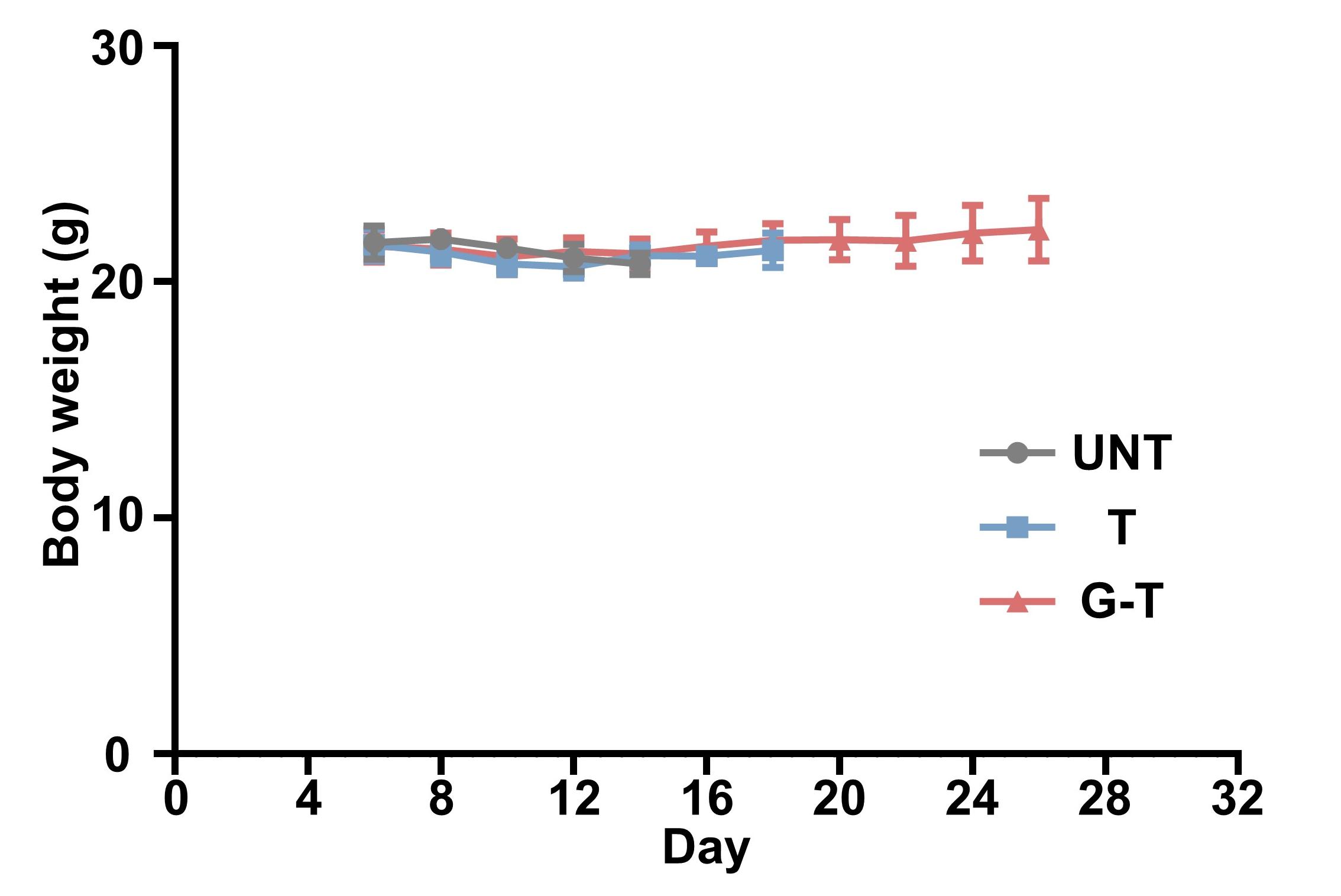


**Figure S23.** Body weight curves of the mice from different groups in the B16-OVA tumor model as indicated. Error bars represent mean ± SD (n = 6).


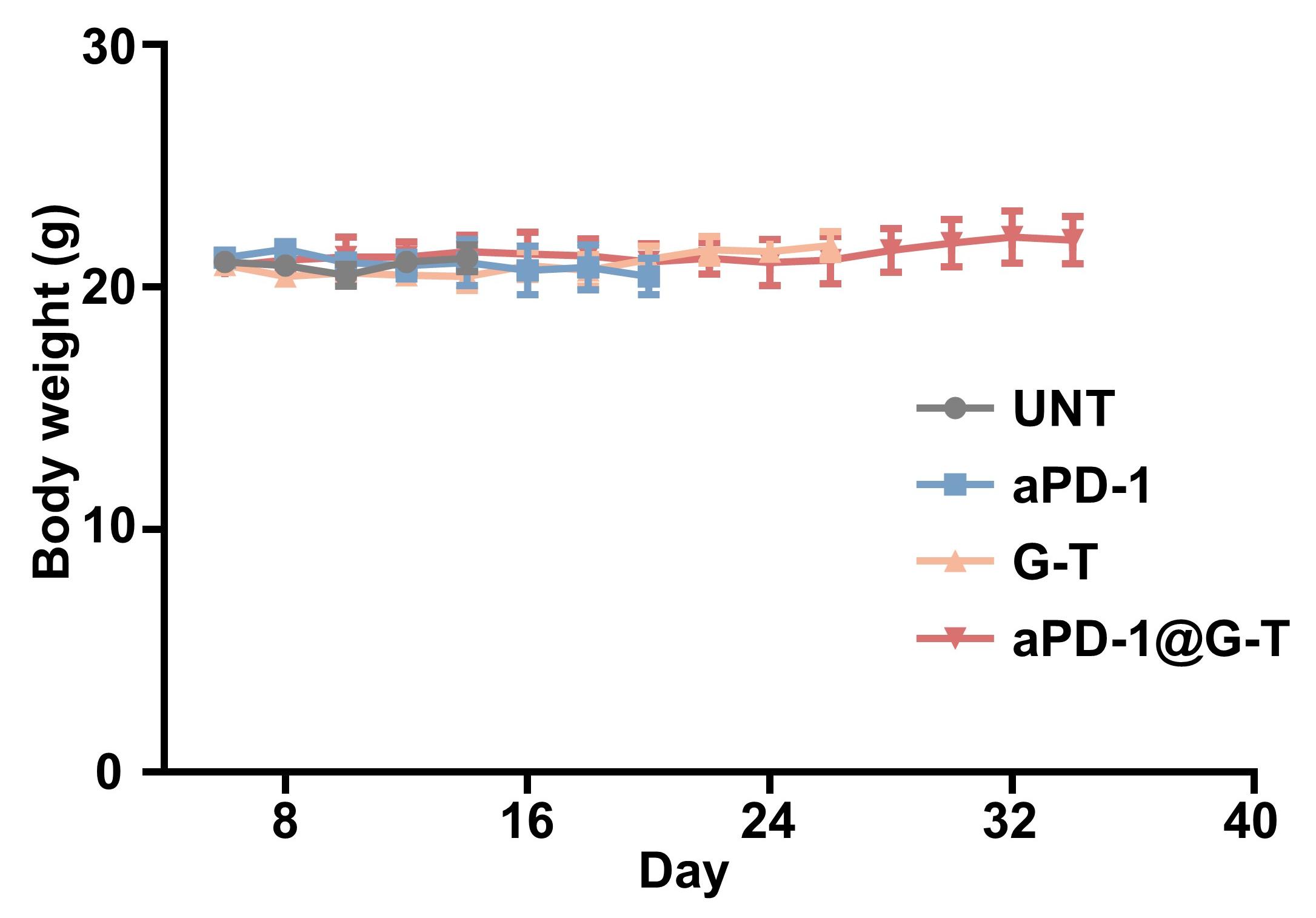


**Figure S24.** Body weight curves of the mice from different groups in the CT-26 tumor model as indicated. Error bars represent mean ±SD (n = 6).
